# Comparative genomics sheds light on the predatory lifestyle of accipitrids and owls

**DOI:** 10.1038/s41598-019-38680-x

**Published:** 2019-02-19

**Authors:** Chuang Zhou, Jiazheng Jin, Changjun Peng, Qinchao Wen, Guannan Wang, Weideng Wei, Xue Jiang, Megan Price, Kai Cui, Yang Meng, Zhaobin Song, Jing Li, Xiuyue Zhang, Zhenxin Fan, Bisong Yue

**Affiliations:** 1 Key Laboratory of Bioresources and Ecoenvironment (Ministry of Education), College of Life Sciences, Sichuan University, Chengdu, 610064 P. R. China; 2 Sichuan Key Laboratory of Conservation Biology on Endangered Wildlife, College of Life Sciences, Sichuan University, Chengdu, 610064 P. R. China

## Abstract

Raptors are carnivorous birds including accipitrids (Accipitridae, Accipitriformes) and owls (Strigiformes), which are diurnal and nocturnal, respectively. To examine the evolutionary basis of adaptations to different light cycles and hunting behavior between accipitrids and owls, we *de novo* assembled besra (*Accipiter virgatus*, Accipitridae, Accipitriformes) and oriental scops owl (*Otus sunia*, Strigidae, Strigiformes) draft genomes. Comparative genomics demonstrated four PSGs (positively selected genes) (*XRCC5*, *PRIMPOL*, *MDM2*, and *SIRT1*) related to the response to ultraviolet (UV) radiation in accipitrids, and one PSG (*ALCAM*) associated with retina development in owls, which was consistent with their respective diurnal/nocturnal predatory lifestyles. We identified five accipitrid-specific and two owl-specific missense mutations and most of which were predicted to affect the protein function by PolyPhen-2. Genome comparison showed the diversification of raptor olfactory receptor repertoires, which may reflect an important role of olfaction in their predatory lifestyle. Comparison of *TAS2R* gene (i.e. linked to tasting bitterness) number in birds with different dietary lifestyles suggested that dietary toxins were a major selective force shaping the diversity of *TAS2R* repertoires. Fewer *TAS2R* genes in raptors reflected their carnivorous diet, since animal tissues are less likely to contain toxins than plant material. Our data and findings provide valuable genomic resources for studying the genetic mechanisms of raptors’ environmental adaptation, particularly olfaction, nocturnality and response to UV radiation.

## Introduction

Recent phylogenetic analyses have classified raptors into three orders: Accipitriformes, Falconiformes, and Strigiformes^1–3^. Eagles and hawks are accipitrids belonging to the order Accipitriformes, which encompasses a large number of species ranging from small hawks to eagles^4^. Hovering and soaring at high altitude is one of the most important techniques employed by accipitrids that hunt in open habitats^5^. Accipitrids are diurnal, and while they are hunting at altitude they are exposed to intense ultraviolet (UV) radiation from sunlight. UV radiation is more intense at high altitude and could consequently cause damage to DNA. However, little is known of the molecular mechanisms that respond to UV radiation and repair damaged DNA in these diurnal accipitrids.

Although birds are primarily diurnal, one important exception is the nocturnal order Strigiformes (owls). The visual system in owls has undergone substantial evolutionary modification to adapt to nocturnality^6^. Owls have large and rod-dominant retinas that are extremely sensitive to light and highly useful in their low light hunting environments^7^. Whereas, diurnal raptors possess color perception and sharp visual acuity in relatively bright environments due to cone-dominant retinas^7^. Although previous studies in morphology, anatomy and behavior have proposed a relationship between nocturnality and functional diversification of low-light visual sensitivity^6–8^, the molecular basis underlying the low-light adaptation of owls is still unclear.

Hunting in many species relies on visual acuity but also on olfaction. Previous reviews have summarized the features of a fully functional avian olfactory system and that many species rely heavily on olfaction^9–11^. Diurnal and nocturnal raptors were previously thought to rely on eyesight for locating prey rather than using olfaction^12^. However, recent studies have reported that some raptors rely more on olfactory cues than visual acuity^13,14^. A previous approach to molecular characterization of the avian olfactory system using PCR (Polymerase Chain Reaction) amplification of olfactory receptor genes (ORs) with degenerate primers^15^ generated an approximate measurement of OR quantity. The quantity of ORs tended to be over-estimated through PCR with degenerate primers and only incomplete fragments were produced^16^. By comparison, *de novo* assembled genomes contributed to a global OR repertoire assessment^17^ and can therefore be employed to obtain a more accurate OR repertoire estimation.

Raptors also rely on other senses to successfully hunt and consume prey, and to avoid unpalatable and toxic food. For example, the detection of bitter tasting food is reported to protect animals from ingesting poisonous substances^18,19^. *TAS2R* genes have been reported as being involved in the animal’s ability to detect bitter and thus potentially poisonous food^20–22^, occurring more frequently in herbivores than in carnivores^23^. Herbivores have undergone a stronger selective pressure to retain *TAS2R* genes because plant tissues are more likely to contain toxins than animal tissues^24^. With an increasing number of raptor genomes, it is meaningful to compare *TAS2R* genes in birds with different dietary lifestyles to determine whether detection of bitterness is important to raptors. Thus, analyses of *TAS2R* variation in select bird species can provide valuable information on the use of taste in hunting raptors, in addition to visual and olfactory cues.

We consequently assembled draft whole-genome sequences of *Accipiter virgatus* (besra) and *Otus sunia* (oriental scops owl) to better understand the evolutionary adaptations related to the predatory lifestyle in diurnal accipitrids and nocturnal owls. We aimed to provide an insight into genomic changes affecting physiological functions (response to UV radiation and DNA damage repair) in diurnal accipitrids and retina development in owls. We annotated ORs in 13 bird species to demonstrate the diversification of raptors’ ORs repertoire. Furthermore, we aimed to compare the *TAS2R* gene number in bird species with varied diets. This work will provide novel insights into the evolutionary history and the genetic basis of adaptations to hunting of diurnal accipitrids and nocturnal owls, and a solid foundation for future raptor genetic and epigenomic studies.

## Results

### Genome sequencing, assembly and quality assessment

Muscle samples from one male *A. virgatus* and one female *O. sunia* were used for genomic sequencing. In total, we generated 168.87 Gb (~143-fold coverage) and 157.04 Gb (~120-fold coverage) of high quality sequences for *A. virgatus* and *O. sunia*, respectively, after filtering out low quality and duplicated reads (Supplementary Tables S1 and S2). We estimated the genome size of *A. virgatus* and *O. sunia* to be 1.18 Gb and 1.30 Gb, respectively, on the basis of K-mer analysis (Supplementary Figs S1 and S2 and Table S3), which was similar to the reported avian genomes (Supplementary Table S4). The total length of all scaffolds was 1.18 Gb and 1.25 Gb, and the scaffold N50 length reached 5.38 Mb and 7.79 Mb for *A. virgatus* and *O. sunia*, respectively (Supplementary Table S5). For genome completeness, CEGMA results showed that 72.98% complete and 88.31% partial gene set for *A. virgatus*, and 83.87% complete and 92.34% partial gene set for *O. sunia* (Supplementary Tables S6 and S7). BUSCO results showed that 81.2% and 90.8% of the eukaryotic single-copy genes were captured for *A. virgatus* and *O. sunia*, respectively. (Supplementary Tables S8 and S9).

### Genome characterization

The GC content of the *A. virgatus* and *O. sunia* genomes were approximately 41.68% and 41.79%, similar to other bird species such as *Gallus gallus* (chicken) and *Taeniopygia guttata* (zebra finch). (Supplementary Fig. S3). We found that about 63.56 Mb and 77.17 Mb sequences (5.37% and 6.19% of the genome assembly) could be attributed to repeats in *A. virgatus* and *O. sunia* genomes, respectively. The percentage of long interspersed nuclear elements (LINEs), long terminal repeats (LTRs), short interspersed nuclear elements (SINEs), and DNA transposons were 2.97%, 1.74%, 0.13%, and 0.52% in *A. virgatus* genome, while 4.33%, 1.21%, 0.12%, and 0.51% in *O. sunia* genome (Supplementary Table S10). Comparison of these four repeat elements in nine raptors demonstrated the dominating role of LINEs in all kinds of repeat elements (Supplementary Figs S4 and S5).

Gene prediction resulted in a total of 16,388 and 15,229 protein-coding genes (PCGs) for *A. virgatus* and *O. sunia* genomes, respectively. The average gene and coding sequence lengths were 27,139/1,665 bp and 29,744/1,851 bp for *A. virgatus* and *O. sunia* genomes. Additionally, *A. virgatus* and *O. sunia* genes had an average of 10 exons and 11 exons per gene, respectively (Supplementary Tables S11 and S12). We found that 14,398 (87.86%) and 14,416 (94.66%) out of 16,388 and 15,299 identified PCGs were well supported by public protein databases (TrEMBL, SwissProt, Nr, InterPro, GO and KEGG) for *A. virgatus* and *O. sunia*, respectively. (Supplementary Figs S6 and S7 and Table S13). In addition, the non-PCGs were annotated: 55 5S rRNA, 200 tRNA, 165 microRNA (miRNA), and 153 snRNA genes for *A. virgatus* (Supplementary Table S14), and 53 5S rRNA, 281 tRNA, 212 microRNA (miRNA), and 207 snRNA genes for *O. sunia* (Supplementary Table S15).

### Bird phylogeny, divergence and evolution of gene families

**Figure 1:**
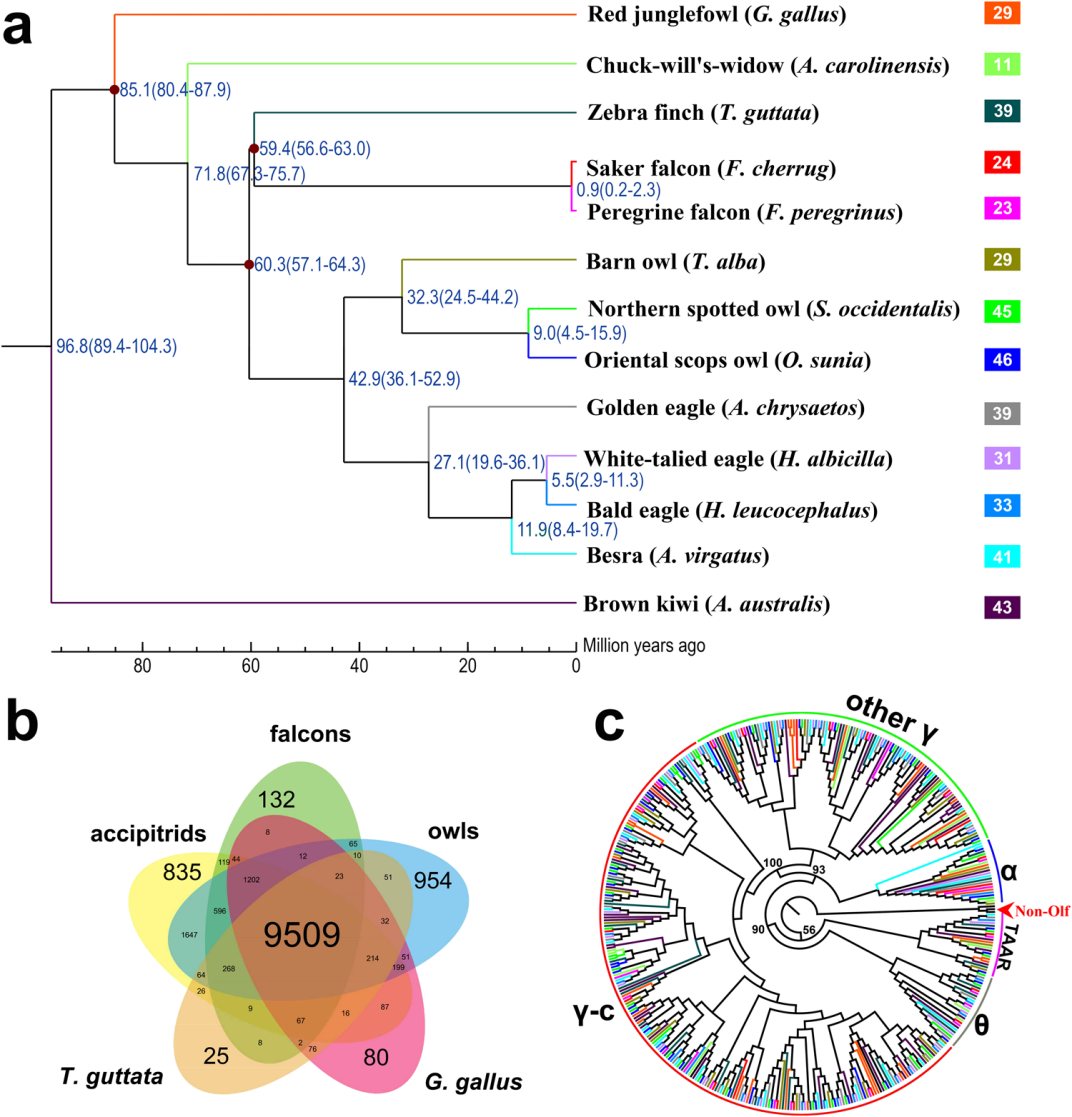
Comparative genomics in avian species studied. **(a)** Phylogenetic tree constructed using 1:1:1 orthologous genes. Branch numbers indicate the number of gene families that have expanded (left) and contracted (right) after the split from the common ancestor. The time lines indicate divergence times among the species. **(b)** Comparison of orthologous gene clusters among accipitrids, falcons, owls, *T. guttata*, and *G. gallus*. **(c)** Maximum likelihood (ML) tree constructed using intact ORs from 13 birds. Three genes (*ADRB1*, *ADRA1A*, and *HTR6*) from family GPCRs were used as outgroup (shown as Non-Olf). ORs of each bird are represented by the same color as the species branch in (**a**). The insets showing the number of intact ORs in each species that are analyzed using ML tree topology were presented behind each species in (**a**).

We identified 16,530 gene families for 13 bird species (four accipitrids: (besra (*A. virgatus*), bald eagle (*Haliaeetus leucocephalus*), white-talied eagle (*Haliaeetus albicilla*), golden eagle (*Aquila chrysaetos*)), three owls (Oriental scops owl (*O. sunia*), barn owl (*Tyto alba*), northern spotted owl (*Strix occidentalis*)), two falcons (peregrine falcon (*Falco peregrinus*), saker falcon (*Falco cherrug*)), zebra finch (*T. guttata*), red junglefowl (*G. gallus*), chuck-will’s-widow (*Antrostomus carolinensis*), and brown kiwi (*Apteryx australis*)) (Supplementary Fig. S8 and Table S16), of which 2,845 represented 1:1 orthologous gene families. Comparison of orthologous gene clusters among accipitrids, falcons, owls, *T. guttata*, and *G. gallus* is showed in Fig. 1b. The maximum likelihood phylogeny constructed based on the 1:1 orthologous genes indicated that owls and accipitrids belong to a subclade that was most likely derived from a common ancestor approximately 42.9 million years ago (Mya) (Fig. 1a). The falcons and other predators diverged 60.3 Mya, and *Falco peregrinus* and *F. cherrug* diverged 0.9 Mya.

### Positive selection in the accipitrid lineage

**Figure 2:**
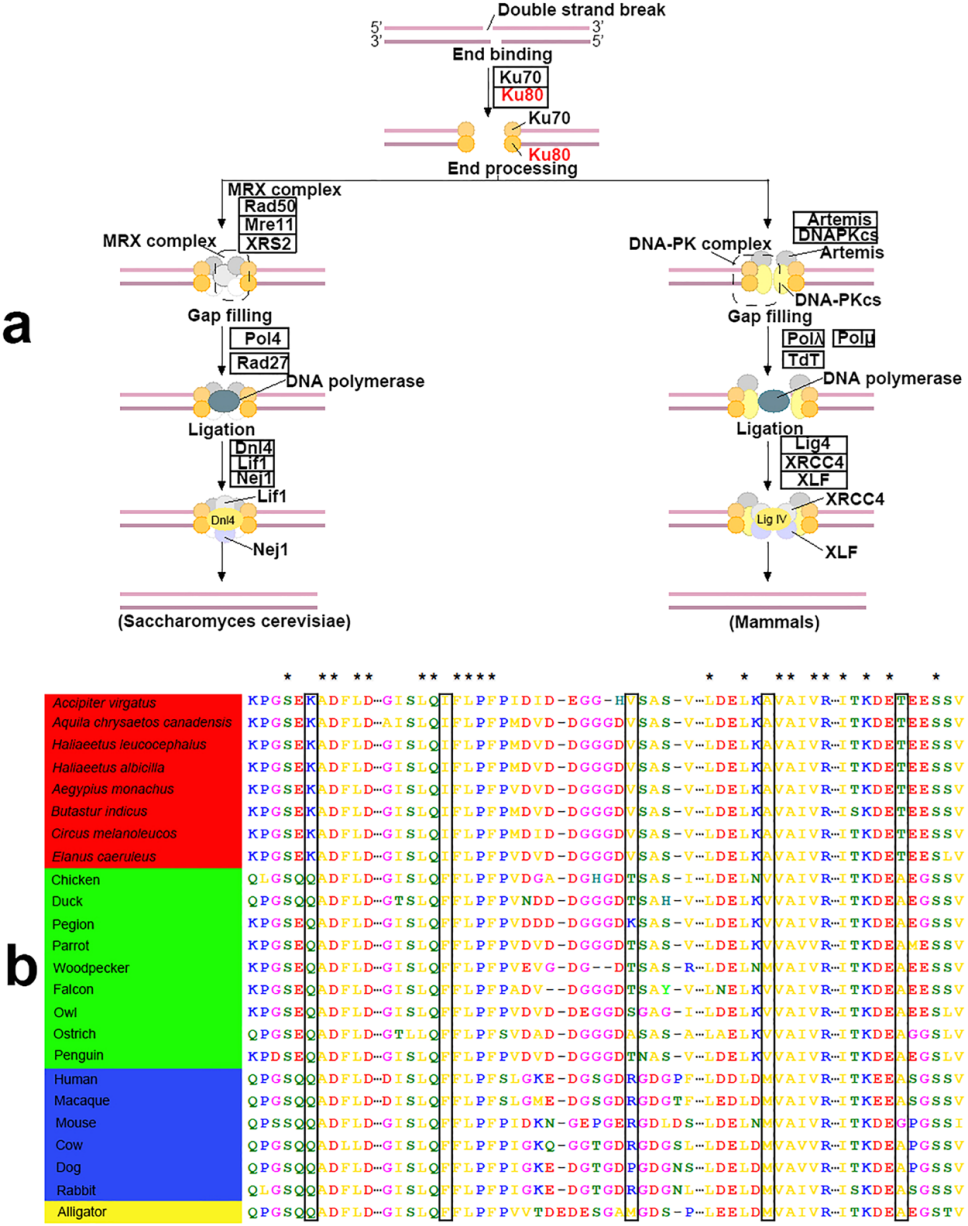
Non-homologous end joining (NHEJ) pathway (KEGG map03450) and multiple amino-acid alignment of *XRCC5*. **(a)** Positively selected gene *XRCC5* (ku80) was shown in red in the NHEJ pathway. **(b)** The missense mutations found in this study were marked within rectangle. The asterisk means all species have the same amino acid type at this position. Species in the red box are accipitrids; species in the green box are other birds; species in the blue box are mammals; species in the yellow box is a reptile.

On the accipitrid branch, we found that 4 genes (i.e. *XRCC5*, *SIRT1*, *PRIMPOL*, *MDM2*) had functional associations with responses to UV radiation and DNA damage repair (Supplementary Table S17). A top gene in our selection scan was *XRCC5*, which was also called ku80. *XRCC5* is important for the repair of DNA ends by non-homologous end joining (NHEJ) (Fig. 2a). Deletion of *XRCC5* in mice has resulted in immune deficiency, growth retardation, increased chromosomal instability, and cancer^25,26^. Altered expression of *XRCC5* has caused oncogenic phenotypes, such as genomic instability, hyper proliferation and resistance to apoptosis, and tumorigenesis^27^, and has been detected in various types of sporadic cancer^28^. We found five missense mutations in *XRCC5* in accipitrids (Fig. 2b), of which three missense mutations were predicted to be deleterious by PolyPhen-2^29^ (Table 1). Further validation showed that all accipitrids had the same amino acid type as *A. virgatus*, *H. leucocephalus*, *H. albicilla* and *A. chrysaetos* at all mutation sites (Fig. 2b). Therefore, it was very clear that the mutations at *XRCC5* were accipitrid-specific.

**Table 1:** Species-specific missense mutations of positively selected genes.

Species	Genes	Mutation sites (human)	Amino acids (raptors/human)	Polarity	PolyPhen-2			
					HumanDiv		HumanVar	
Accipitrids	*XRCC5*	104	K(Lys)/Q(Gln)	polar/polar	0.27	benign	0.055	benign
		163	I(Ile)/F(Phe)	unpolar/unpolar	0.89	possibly damaging	0.557	possibly damaging
		178	V(Val)/R(Arg)	unpolar/polar	0.047	benign	0.083	benign
		389	A(Ala)/M(Met)	unpolar/unpolar	0.937	possibly damaging	0.967	probably damaging
		691	T(Thr)/A(Ala)	polar/unpolar	0.919	possibly damaging	0.312	benign
Owls	*ALCAM*	259	I(Ile)/A(Ala)	unpolar/unpolar	0.617	possibly damaging	0.336	benign
		297	M(Met)/T(Thr)	unpolar/polar	0.85	possibly damaging	0.557	possibly damaging

In response to ionizing radiation, *SIRT1* was reported to play a vital part in DNA repair^30,31^. *SIRT1* null MEF cells were observed to be more sensitive than control MEF cells in response to UV irradiation, indicating that *SIRT1* was possibly involved in UV-induced DNA repair^32^. Murine double minute 2 (*MDM2*) was an important component of the response to UV radiation, and cells with decreased levels of *MDM2* showed more sensitivity to ionizing radiation^33^. *PRIMPOL* played a critical role in damage tolerance to UV irradiation during DNA replication^34,35^. Decreased levels of *PRIMPOL* sensitized mammalian and avian cells to UV irradiation^36–38^, suggesting that *PRIMPOL* was vital for recovery from UV damage.

### Positive selection on the owl branch

**Figure 3:**
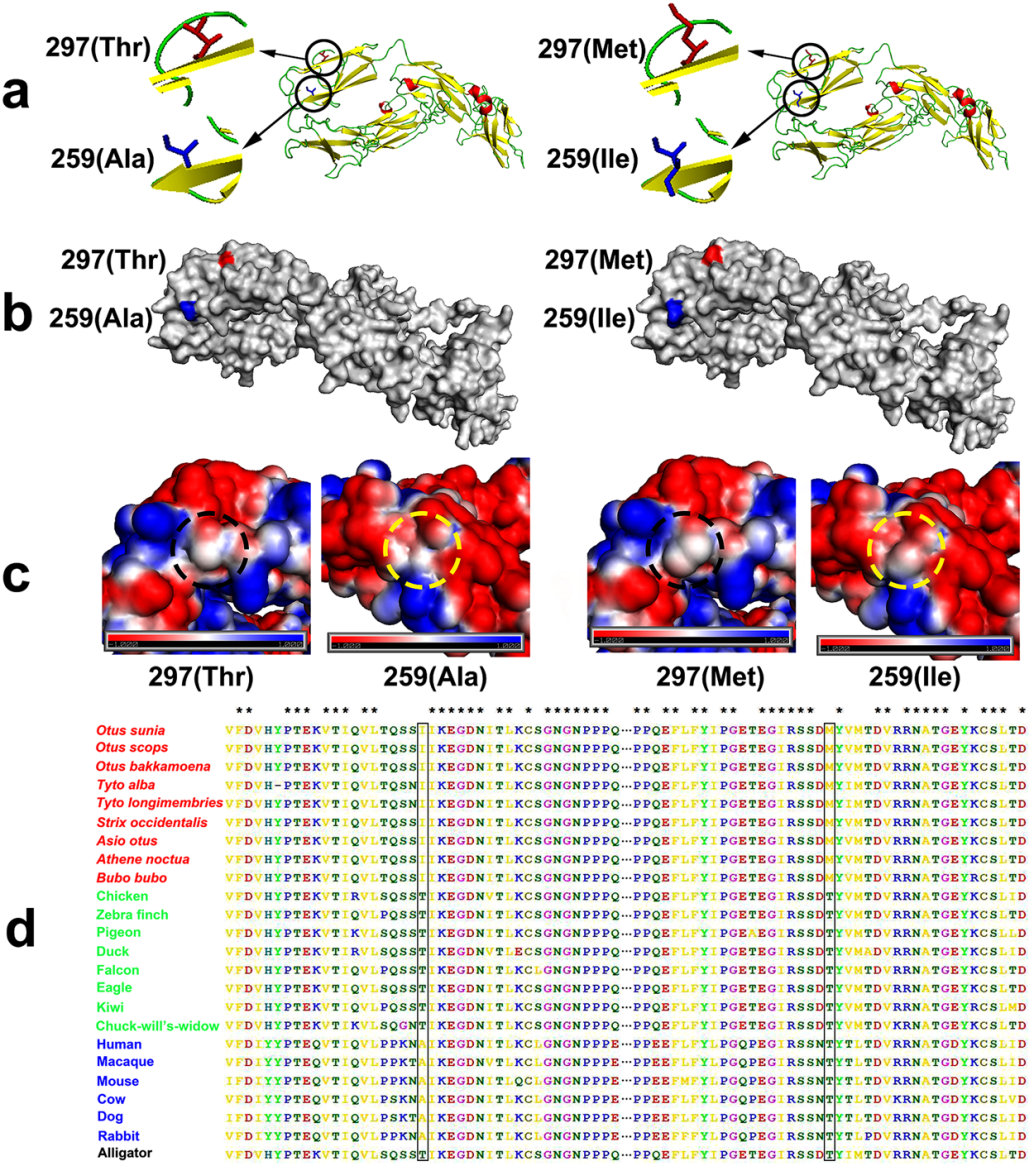
Amino-acid sequence alignment of *ALACM* and three kinds of visualization of non-mutated and mutated *ALACM*. **(a)** Altered amino acids at p259 and p297 are shown in non-mutant and mutant *ALCAM* protein models. **(b)** In the surface of non-mutant and mutant *ALCAM*, mutation sites of p259 and p297 are colored as blue and red, respectively. **(c)** Electrostatic potential maps on the surface of p259 and p297 residues. Compared with non-mutant *ALCAM*, the p259 mutation in mutant *ALCAM* shows a trend of negatively charged region, while p297 mutation tends to be neutral (blue: positive charges; red: negative charges). **(d)** Two missense mutations in *ALCAM* were marked within rectangle. The asterisk means all species have the same amino acid type at this position. Species in red are owls; species in green are other birds; species in blue are mammals; species in black is a reptile.

We found one PSG, *ALCAM*, had direct correlation with retina development in owls (Supplementary Table S17). It has been documented that *ALCAM* can guide retinal axons in *Drosophila* and rodents^39,40^, and *ALCAM* has a potential role in human retinal angiogenesis^41^. Angiogenesis is a crucial mechanism in ischemic retinal vasculopathy pathogenesis^42^, and previous studies have demonstrated that *ALCAM* might participate in both processes^41^. The determination of *ALCAM*’s involvement in these processes is important as ischemic retinal vasculopathies and posterior uveitis have led to vision loss in people in the United States and worldwide^43,44^. Two owl-specific missense mutations were identified in *ALCAM* (Fig. 3d), and both missense mutations were predicted to affect the protein function by PolyPhen-2 (Table 1). Further validation, including PCR confirmed that all the owls had the same amino acid type as *O. sunia*, *T. alba* and *S. occidentalis* at both mutation sites, which demonstrated that both mutations at *ALCAM* were owl-specific. Both missense mutations had a deleterious influence on protein structure (Fig. 3a–c).

### Olfactory Receptor Genes (ORs)

We annotated the ORs in 13 bird species based on putative functionality and seven transmembrane helices (7TM) (Supplementary Table S18). Generally, the number of ORs in raptors varied between orders: owls had the most, accipitrids medium, and falcons least. Overall, the average number of ORs in raptors was not less than in other bird species. Comparative analyses of the OR repertoire showed that most ORs in avian genomes were the γ subgroup of type 1 OR genes, in accordance with previously sequenced avian genomes^15^. Phylogenetic comparison of OR repertoires suggested that γ ORs in birds did not show an obvious species-specific clustering pattern, which was different from previous studies^45^. The gene family size of ORs in this study was very likely to have directly caused this contrast. Finally, a few γ ORs of the kiwi were basal to many clades containing γ ORs (Fig. 1c).

### Dietary Lifestyle

**Figure 4:**
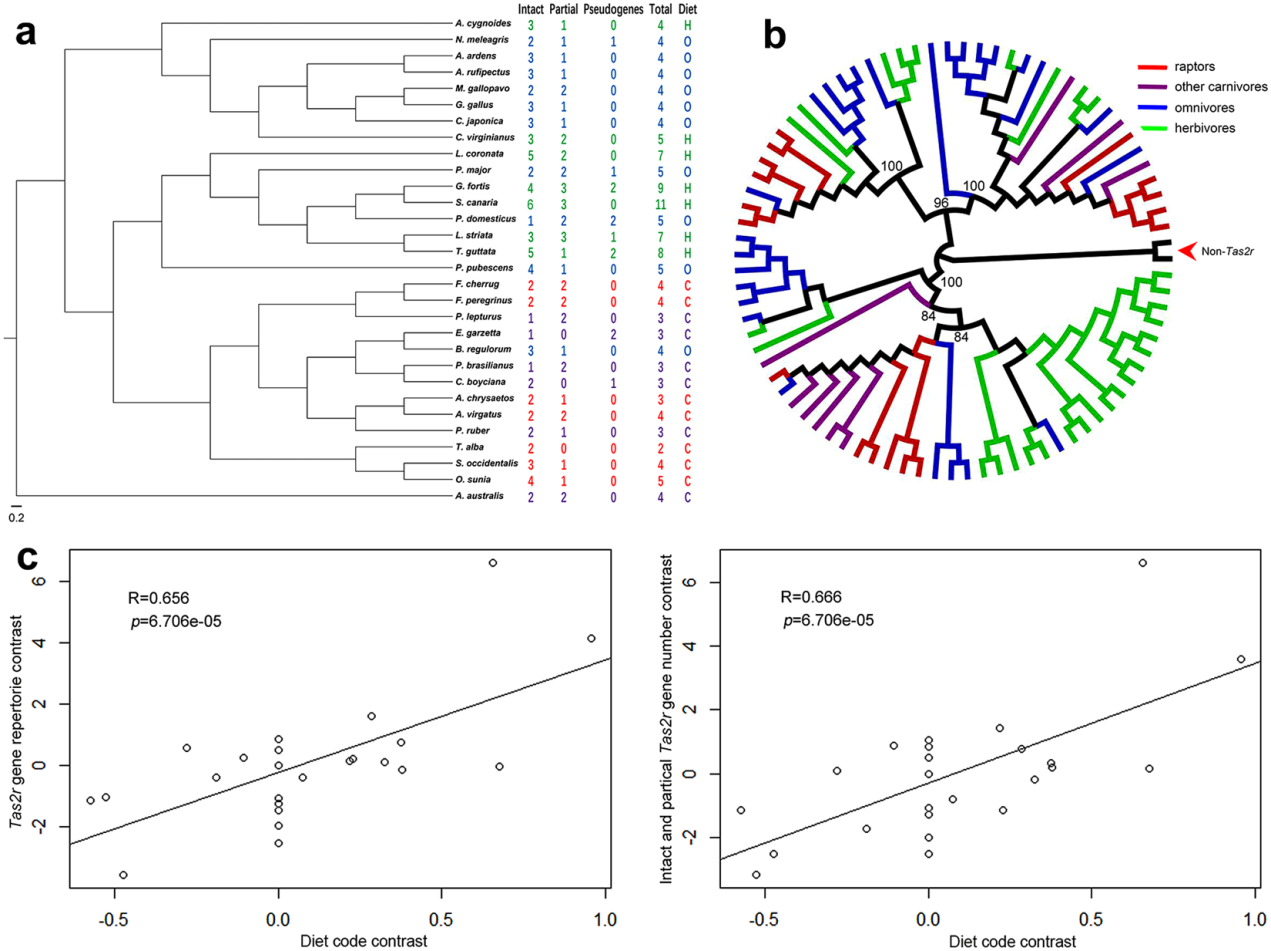
Phylogenetic analyses of *TAS2R*s and the impacts of diet to *TAS2R* repertoire size. **(a)** The *TAS2R* gene repertoires of 30 birds identified in this study. The sources of dietary information were shown in Supplementary Table S22. C, carnivorous; H, herbivorous; O, omnivorous. **(b)** Evolutionary relationships of all 81 intact *TAS2R* genes from 30 birds. **(c)** PIC in a total number of *TAS2R*s (intact genes, partial genes, and pseudogenes) was positively correlated with PIC in diet codes significantly. PIC in a total number of *TAS2R*s (intact genes and partial genes) was positively correlated with PIC in diet codes significantly. The diet code is 0, 0.5, and 1 for the carnivorous birds, the omnivorous birds, and the herbivorous birds, respectively.

The total number of *TAS2R* genes of accipitrids, falcons, and owls varied little (Fig. 4a). There were no pseudogenes in the *TAS2R* repertoires in all raptors we studied, and the percentage of pseudogenes in birds was much lower than other vertebrates reported^23^. Compared to carnivorous birds, omnivorous birds tended to possess more *TAS2R* genes, while herbivorous birds possessed the most. It was reported that some *TAS2R* lineages were enriched because of species-specific gene duplications, and other lineages were relatively duplication free^46^. This phenomenon was also obvious in the phylogenetic tree we constructed (Fig. 4b). The lineages, which were marked with one color, demonstrated a cluster of genes belonging to the same species or group of closely related species. The lineages marked with many colors illustrated genes from distantly related species. There was positive correlation between the phylogenetically independent contrasts (PICs) in the dietary code and the PICs in the *TAS2R* gene repertoire size (R = 0.656, P = 6.706e-05) (Fig. 4c). Equally, the positive correlation was verified while discarding pseudogenes (R = 0.666, P = 4.859e-05) (Fig. 4c), and the difference between both correlations was not significant.

## Discussion

The evolution of genes associated with the response to UV radiation and DNA damage repair may have helped diurnal raptors cope with flying or soaring at high altitudes. This type of adaptation has also been developed by other animals living at high altitude^47^. We identified four genes that are under positive selection in the accipitrid lineage, which were *XRCC5*, *MDM2*, *PRIMPOL*, and *SIRT1*, and conformed to this adaptation strategy. *XRCC5* had five accipitrids-specific mutations that possibly affected the protein function. This finding may be related to the increased exposure of accipitrids to UV radiation compared to non-high-altitude birds. It is possible that potentially important reorganization of the radiation resistance system has taken place in the diurnal raptors since their divergence from other bird species, which may be related to the diurnal raptor lifestyle or ecology.

There was a substantial evolutionary modification in the owl visual system^6^ due to nocturnal adaptation. Owls possess large and rod-dominant retinas that are extremely sensitive to light, providing them with extraordinary night vision^6^. We found a PSG (*ALCAM*) associated with retina development, which also had two owls-specific mutations that possibly affected the protein function. This PSG possibly enhances low-light sensitivity, and thus resulted in high visual acuity at night. This gene and gene family expansions related to photoreceptor differentiation or development and retina development possibly worked together to enhance owl nocturnality.

It has long been hypothesized that raptors rely on eyesight for locating prey^12^. We found that the number of ORs in raptors was not less than other studied bird species, suggesting that raptors possibly have similar levels of olfactory sense. Our finding of the dual use of olfaction and vision in raptors is consistent with previous studies^13,14^.

It has been reported that each *TAS2R* gene can discern different bitter compounds and different *TAS2R* genes have contrasting sensitivities to the same bitter compounds^48^. Previous studies assumed that *TAS2R* gains through gene duplication could raise the number of detectable toxins, while the losses of *TAS2R*s would decrease this number^23^. Thus, the herbivores were likely to have more functional *TAS2R* genes than the carnivores. In this study, the carnivorous birds possessed the least *TAS2R* genes, while the herbivorous birds the most. Despite our findings being congruent with previous studies, the limited number of avian species and the qualitative dietary codes for species analyzed restricted the statistical power of our analyses, and therefore more species and quantitative dietary information should be employed in the future. Since *TAS2R* genes are capable of detecting different bitter compounds^48^, a variety of *TAS2R* genes should be included in future studies.

In summary, this is the first report describing the complete *Otus* genome and *Accipiter* genome. Comparative genomics confirmed the PSGs associated with the response to UV radiation and DNA damage repair were present in diurnal raptors while not in nocturnal raptors and other bird species. Furthermore, the diversification of OR repertoires highlighted the importance of olfaction in the predatory lifestyle of raptors. We also confirmed that the genomic changes present in the owl genomes enhanced nocturnal vision. Analyses of *TAS2R* gene number in birds with different dietary lifestyles indicated that the *TAS2R* repertoire diversity was chiefly determined by feeding habits. Our *de novo* assembled genomes presented here will provide a resource for the future examination of evolution and adaptation of raptors to their environment and diet, and the genomes will eventually be the useful reference in aiding the long-term conservation of raptors and their genetic diversity, such as the investigation of the evolutionary adaptation and polymorphic microsatellite loci development.

## Methods

### Sampling and sequencing

Muscle samples of a wild male *A. virgatus* and a wild female *O. sunia* that both died of natural causes, were collected from Laojunshan National Nature Reserve (Yibin, Sichuan Province, China). Collected muscle samples were used for genomic DNA extraction, isolation and sequencing. We used a whole genome shotgun approach on the Illumina HiSeq. 2000 platform to sequence the genome. We constructed two paired-end libraries with insert sizes of 230 base pairs (bp) and 500 bp, and three mate-paired libraries with insert sizes of 2 kb, 5 kb and 10 kb.

### Genome size estimation, genome assembly and completeness assessment

Before assembly, a 17-Kmer analysis was performed for estimating the genome size of *A. virgatus* and *O. sunia* genomes with 230 bp libraries, respectively. The assemblies were first performed by SOAPdenovo2^49^ with the parameters set as “all -d 2 –M 2 –k 35”. After using SSPACE^50^ to build super-scaffolds, Intra-scaffold gaps were then filled using Gapcloser with reads from short-insert libraries. CEGMA^51^ and BUSCO^52^ were used to evaluate the genome completeness.

### Gene prediction and annotation

We combined the *de novo* and homology-based prediction to identify PCGs in *A. virgatus* and *O. sunia*. The *de novo* prediction was performed on the assembled genomes with repetitive sequences masked as “N” based on the HMM (hidden Markovmodel) algorithm. AUGUSTUS^53^ and GENSCAN^54^ programs were executed to find coding genes, using appropriate parameters. For the homology prediction, proteins of *G. gallus*, *F. cherrug*, *F. peregrinus*, and humans were mapped onto both genomes using TblastN^55^ with an E-value cutoff of 1E-5. To obtain the best matches of each alignment, the results yielded from TblastN were processed by SOLAR^56^. Homologous sequences were successively aligned against the matching gene models using GeneWise^57^. We used EVidenceModeler (EVM)^58^ to integrate the above data and obtained a consensus gene set. We used Repeatmasker^59^ to identify the repetitive sequences in the genomes of *A. virgatus* and *O. sunia*. tRNA genes were identified by tRNAscan-SE^60^. *A. virgatus* and *O. sunia* genomes were aligned against the rRNAs database to identify rRNA with blastN. We used INFERNAL^61^ by searching against the Rfam database with default parameters to identify the other ncRNAs, including miRNA and snRNA.

### Functional annotation

Functional annotation of the *A. virgatus* and *O. sunia* genes was undertaken based on the best match derived from the alignments to proteins annotated in SwissProt and TrEMBL databases^62^. Functional annotation used BLASTP tools with the same E-value cut-off of 1E-5. Descriptions of gene products from Gene Ontology (GO) ID were retrieved based on the results of SwissProt. We also annotated proteins against the NCBI non-redundant (Nr) protein database. The motifs and domains of genes were annotated using InterProScan^63^ against publicly available databases, including ProDom^64^, PRINTS^65^, PIRSF^66^, Pfam^67^, ProSiteProfiles^68^, PANTHER^69^, SUPERFAMILY^70^, and SMART^71^. To find the best match and involved pathway for each gene, all genes were uploaded to KAAS^72^, a web server for functional annotation of genes against the manually corrected KEGG genes database by BLAST, using the bi-directional best hit (BBH) method.

### Analyses of gene family, phylogeny, and divergence

We used orthoMCL^73^ to define orthologous genes from 13 avian genomes (Supplementary Table S19). Phylogenetic analyses of these 13 birds were constructed using 1:1 orthologous genes. Coding sequences from each 1:1 orthologous family were aligned by PRANK^74^ and concatenated to one sequence for each species for building the tree. Modeltest (ver. 3.7) was used to select the best substitution model^75^. RAxML^76^ was then applied to reconstruct ML phylogenetic trees with 1,000 bootstrap replicates. Divergence time estimation was performed by PAML MCMCTREE^77^.

### Positive selection analyses

The above alignments of 1:1 orthologous genes and phylogenetic tree were used to estimate the ratio of the rates of non-synonymous to synonymous substitutions (ω) per gene by ML with the codeml program within PAML^77^ under the branch-site model. We then performed a likelihood ratio test and identified the PSGs of the accipitrid and owl branches, respectively.

### Validation of the species-specific missense mutations with protein sequences obtained from Genbank

In order to validate the species-specific missense mutations in genes mentioned above, we downloaded all available protein sequences of birds, 6 mammals (human, macaque, mouse, cow, dog, and rabbit), and one reptile (alligator) of each gene from Genbank. All these protein sequences, together with protein sequences identified in genomes assembled in this study, were aligned using MEGA7^78^ for each gene to validate the species-specific missense mutations.

### Transcriptome assembly and gene identification for validating the species-specific missense mutations

To verify the species-specific missense mutations, we downloaded the transcriptome sequencing data of accipitrids (*Aegypius monachus*: SRR3203236, *Butastur indicus*: SRR3203233, *Circus melanoleucos*: SRR3203217, and *Elanus caeruleus*: SRR3203227), and owls (*Otus scops*: SRR3203230, *Otus bakkamoena*: SRR3203243, *Tyto longimembris*: SRR3203222, *Asio otus*: SRR3203220, *Athene noctua*: SRR3203242, and *Bubo bubo*: SRR3203225) from Genbank. The RNA-seq reads were *de novo* assembled into contigs using Trinity^79^ (Grabherr *et al*. 2011). Based on the Trinity results, we identified genes (*XRCC5* and *ALCAM*) using TBLASTN.

### PCR amplification and sequencing for validating the species-specific mutations

Due to the sample availability, we only conducted PCR validation for the owl-specific mutations. Twenty muscle samples of owls (Supplementary Table S20) were collected from the Museum of Sichuan University to verify the owl-specific missense mutation sites in gene *ALCAM*. Primers (Supplementary Table S21) were designed by comparing gene sequences of *G. gallus*, *T. guttata*, *A. virgatus*, *F. peregrinus*, and *O. sunia* with Primer Premier 5^80^. The amplification of genes was carried out with TaKaRa RTaq (TaKaRa Biomedical, Japan) and implemented on a PTC-100 thermal cycler (BioRad, Hercules, CA) in the reaction mixture. The PCR products were sequenced on an ABI PRISM 3730 DNA sequencer in Tsingke Biotechnology Company (Chengdu, Sichuan Province, China) after electrophoresing in 2% agarose gel.

### Protein structure determination

The crystal structure of *ALCAM* was obtained from SWISS-MODEL^81^. We converted the PDB files to PQR format with PDB2PQR server^82^. The PDB files were used for visualization of cartoon and surface representations of gene mutants. The visualization of the electrostatic surface potential was conducted using the APBS plugin in PyMOL^83^.

### Analyses of olfactory receptor genes (ORs)

We constructed an OR database based on reference OR protein sequences downloaded from uniprot^84^. Thirteen studied avian genomes were aligned to the ORs database constructed above with TBLASTN. According to the results, we confirmed the intact olfactory receptor genes by a series of steps^85^. The OR gene repertoire estimated above and three non-ORs downloaded from Genbank were used for comparative phylogenetic analyses. The phylogenetic tree was constructed using the neighbor-joining method implemented in MEGA7. The reliability of the phylogenetic trees was evaluated with 1,000 bootstrap replicates.

### Analyses of *TAS2R* genes

*TAS2R* database was constructed based on reference *TAS2R* protein sequences downloaded from uniprot^84^. The genomes of 10 omnivorous birds, 7 herbivorous birds, 7 studied raptors, and another 6 carnivorous birds were aligned to the *TAS2R* database constructed above with TBLASTN (Supplementary Table S22). We followed a previous study^23^ in identifying *TAS2R* genes. A neighbor-joining tree of 81 protein sequences of intact *TAS2R*s was constructed using MEGA7 with Poisson-corrected gamma distances. The reliability of the estimated tree was evaluated by the bootstrap method with 1,000 bootstrap replications. The package Analyses of Phylogenetics and Evolution^86^ was used to perform the PIC analyses^87^, and the tree used in this analysis was built with 12 mitochondrial PCGs via RAxML with 1,000 bootstraps.

## Supplementary information

Supplementary information

## Data Availability

Genome and DNA sequencing data of besra and oriental scops owl have been deposited into the NCBI Sequence Read Archive (SRA) under the BioProject ID PRJNA420185. All other data supporting the findings of this study are available in the article and its Supplementary Material or are available from the authors upon request. More detailed information for the approaches has been revealed in the Supplementary Material.
