# Supplementary material for: Comparative genomics sheds light on the predatory lifestyle of accipitrids and owls
Source: Sci Rep. 2019 Feb 19;9:2249. doi: 10.1038/s41598-019-38680-x (PMC6381159; doi:10.1038/s41598-019-38680-x)
Supplement: Supplementary file 1 — Supplementary information [file 41598_2019_38680_MOESM1_ESM.docx]

**Supplementary information**

**Comparative genomics sheds light on the predatory lifestyle of accipitrids and owls**

Chuang Zhou^1^, Jiazheng Jin^1^, Changjun Peng^1^, Qinchao Wen^2^, Guannan Wang^1^, Weideng Wei^1^, Xue Jiang^2^, Megan Price^1^, Kai Cui^1^, Yang Meng^1^, Zhaobin Song^2^, Jing Li^1^, Xiuyue Zhang^2^, Zhenxin Fan^2^, Bisong Yue^1^

^1^ Key Laboratory of Bioresources and Ecoenvironment (Ministry of Education), College of Life Sciences, Sichuan University, Chengdu, 610064, P.R. China

^2^ Sichuan Key Laboratory of Conservation Biology on Endangered Wildlife, College of Life Sciences, Sichuan University, Chengdu, 610064, P.R. China

Correspondence and requests for materials should be addressed to B.Y. (email: bsyue@scu.edu.cn) or Z.F. (email: zxfan@scu.edu.cn).

Corresponding author: Bisong Yue, Key Laboratory of Bio-resources and Eco-environment (Ministry of Education), College of Life Sciences, Sichuan University, Chengdu, 610064, P.R. China.

Tel: + 86 28 85412057. Fax: + 86 28 85414886.

Email: [bsyue@scu.edu.cn](mailto:bsyue@scu.edu.cn)

Corresponding author: Zhenxin Fan, Sichuan Key Laboratory of Conservation Biology on Endangered Wildlife, College of Life Sciences, Sichuan University, Chengdu, 610064, P.R. China.

Email: [zxfan@scu.edu.cn](mailto:zxfan@scu.edu.cn)

Chuang Zhou and Jiazheng Jin contributed equally to this work.

The supplementary file contains:

Supplementary Figures 1-8

Supplementary Tables 1-25

Supplementary Note

Supplementary references

# Supplementary Figures


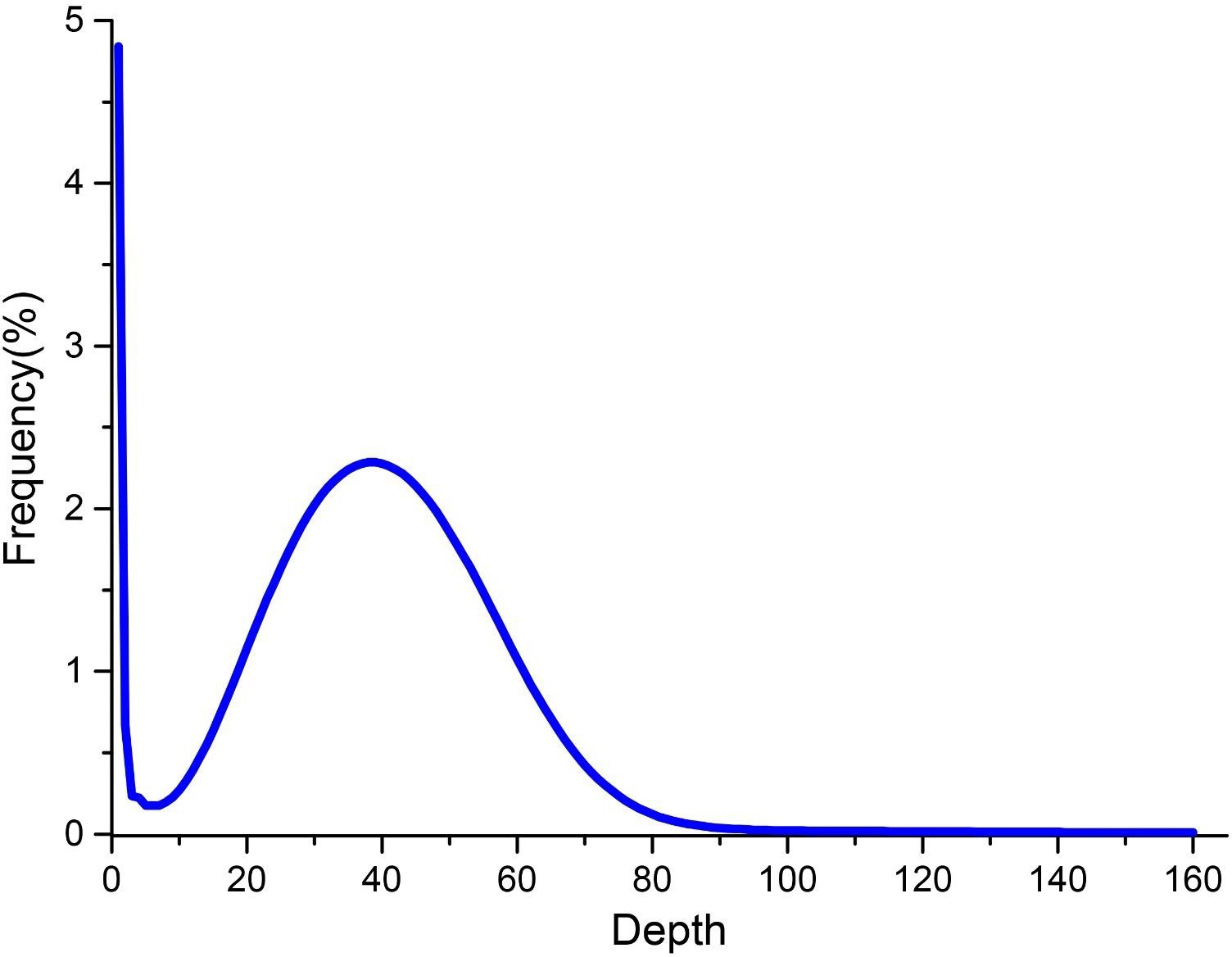


**Supplementary Fig. S1** The distribution of 23-mer frequency in *A. virgatus* genome. The X-axis represents the sequencing depth and the Y-axis represents the ratio of K-mer counts in total K-mers. The frequency of each K-mer follows a Poisson distribution in a given data set. The short-insert-size library (230 bp) after quality control was used to generate the 23-mer sequences. The peak depth was 39 and the genome size was estimated to be 1.18 Gb.


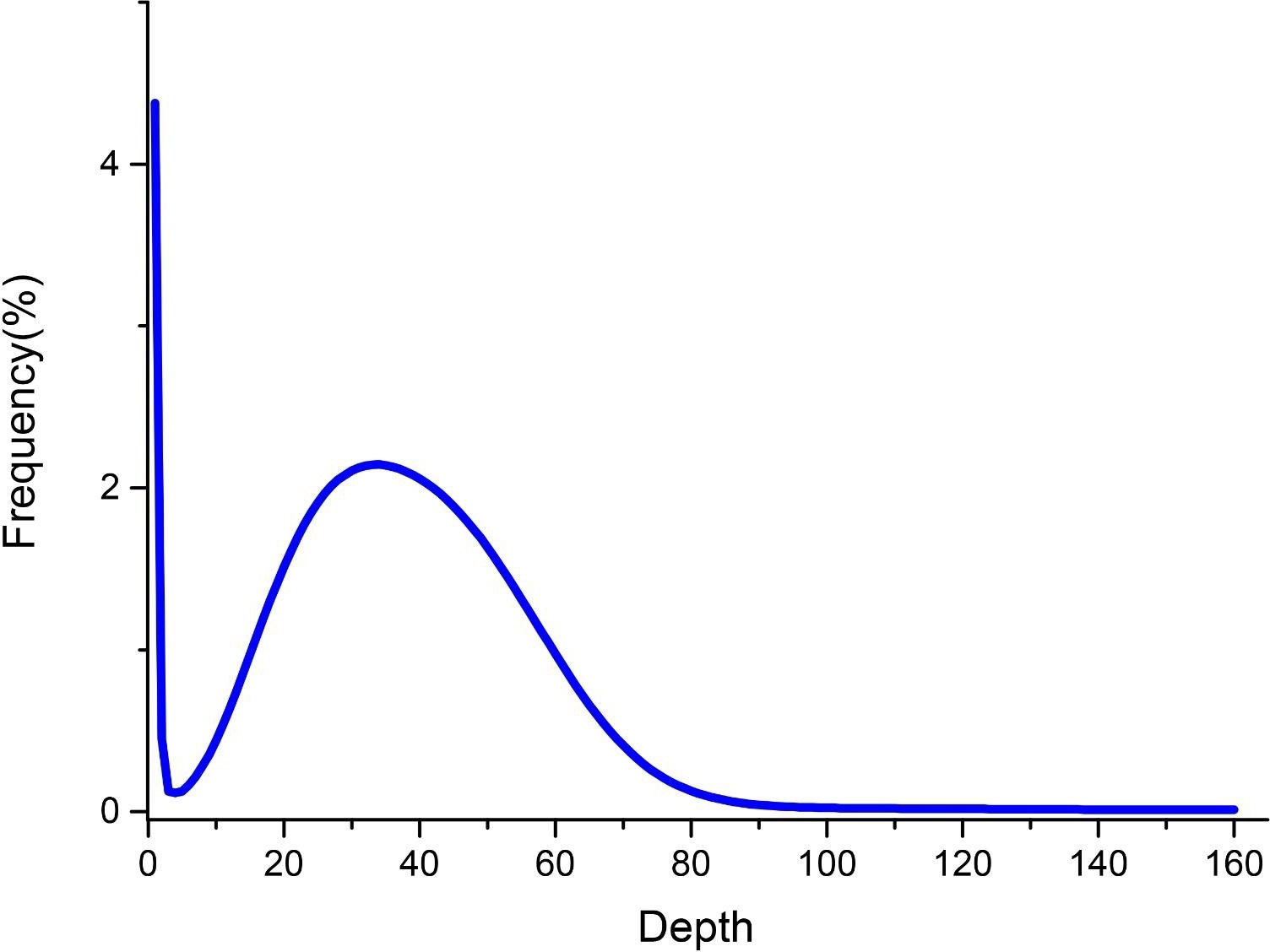


**Supplementary Fig. S2** The distribution of 23-mer frequency in *O. sunia* genome. The X-axis represents the sequencing depth and the Y-axis represents the ratio of K-mer counts in total K-mers. The frequency of each K-mer follows a Poisson distribution in a given data set. The short-insert-size library (230 bp) after quality control was used to generate the 23-mer sequences. The peak depth was 34 and the genome size was estimated to be 1.30 Gb.


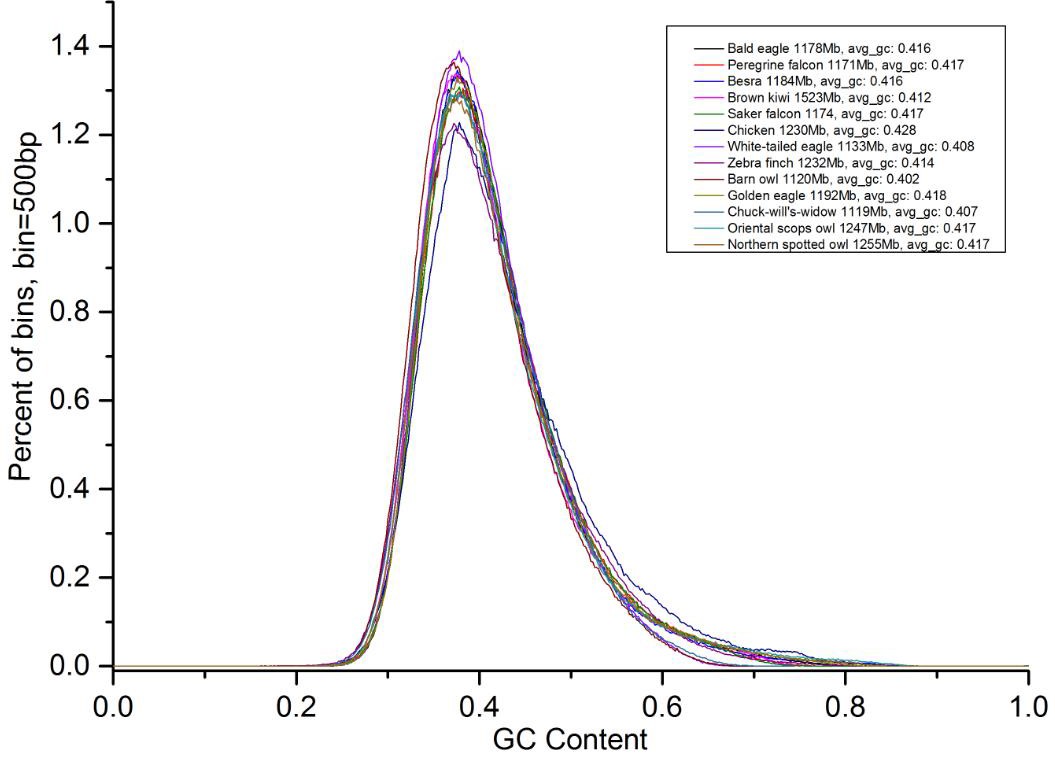


**Supplementary Fig. S3** GC content distributions of 13 avian genomes. The X-axis represents GC content and the Y-axis represents the ratio of GC content in total amount. We downloaded 13 avian genomes and used 500 bp sliding windows to calculate GC content. The GC content of these 13 bird species is similar, between 0.402 and 0.428.


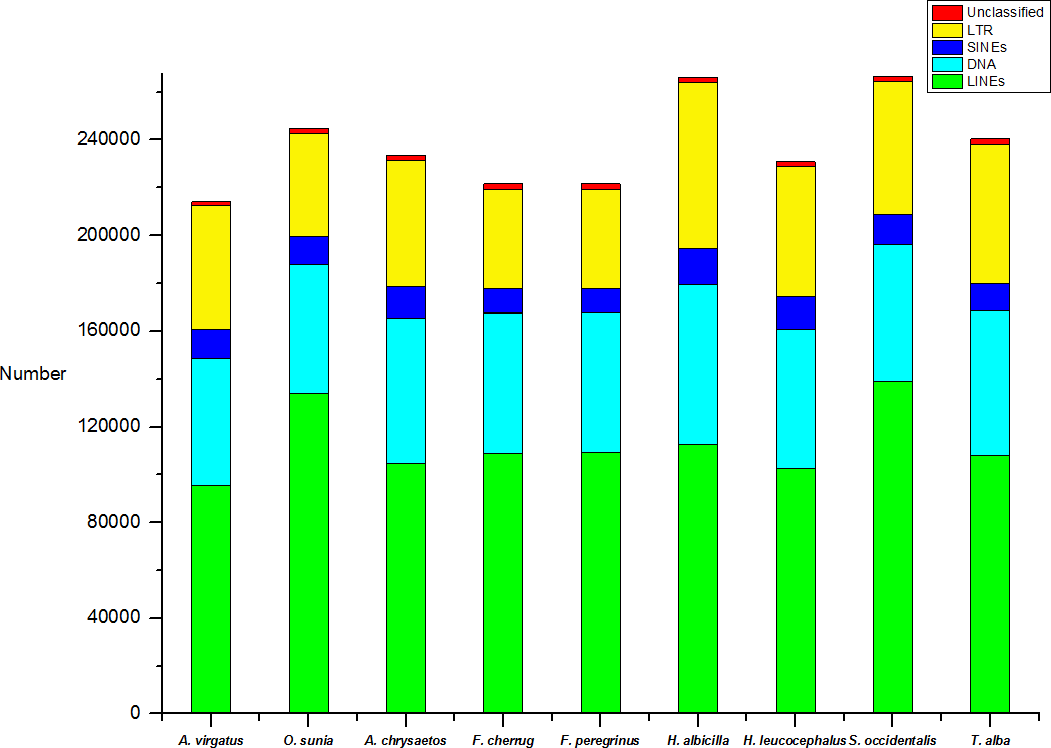


**Supplementary Fig. S4** Number comparison of repeat elements in nine raptors.


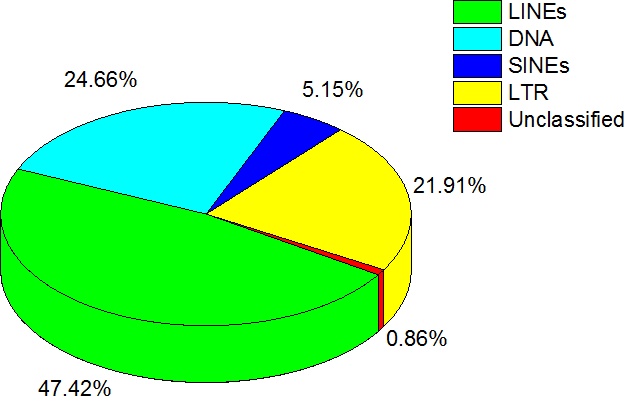


**Supplementary Fig. S5** Average percentage of several repeat elements in nine birds of prey.


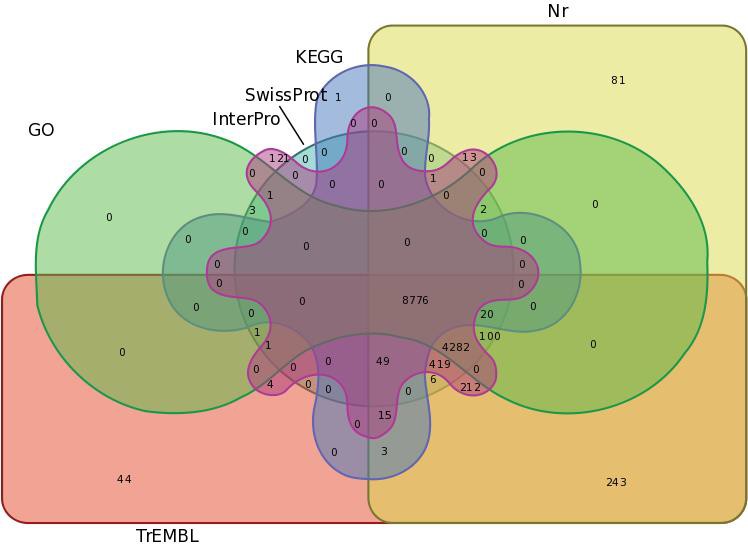


**Supplementary Fig. S6** Functional annotation of *A. virgatus* genome.


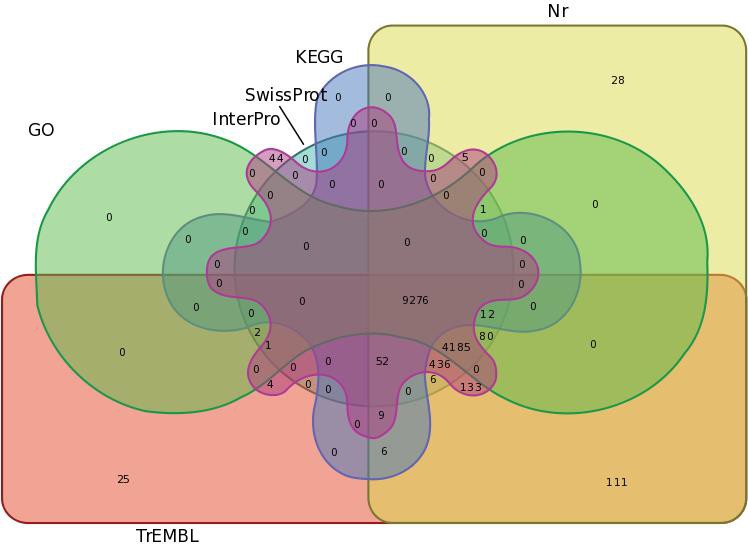


**Supplementary Fig. S7** Functional annotation of *O. sunia* genome.


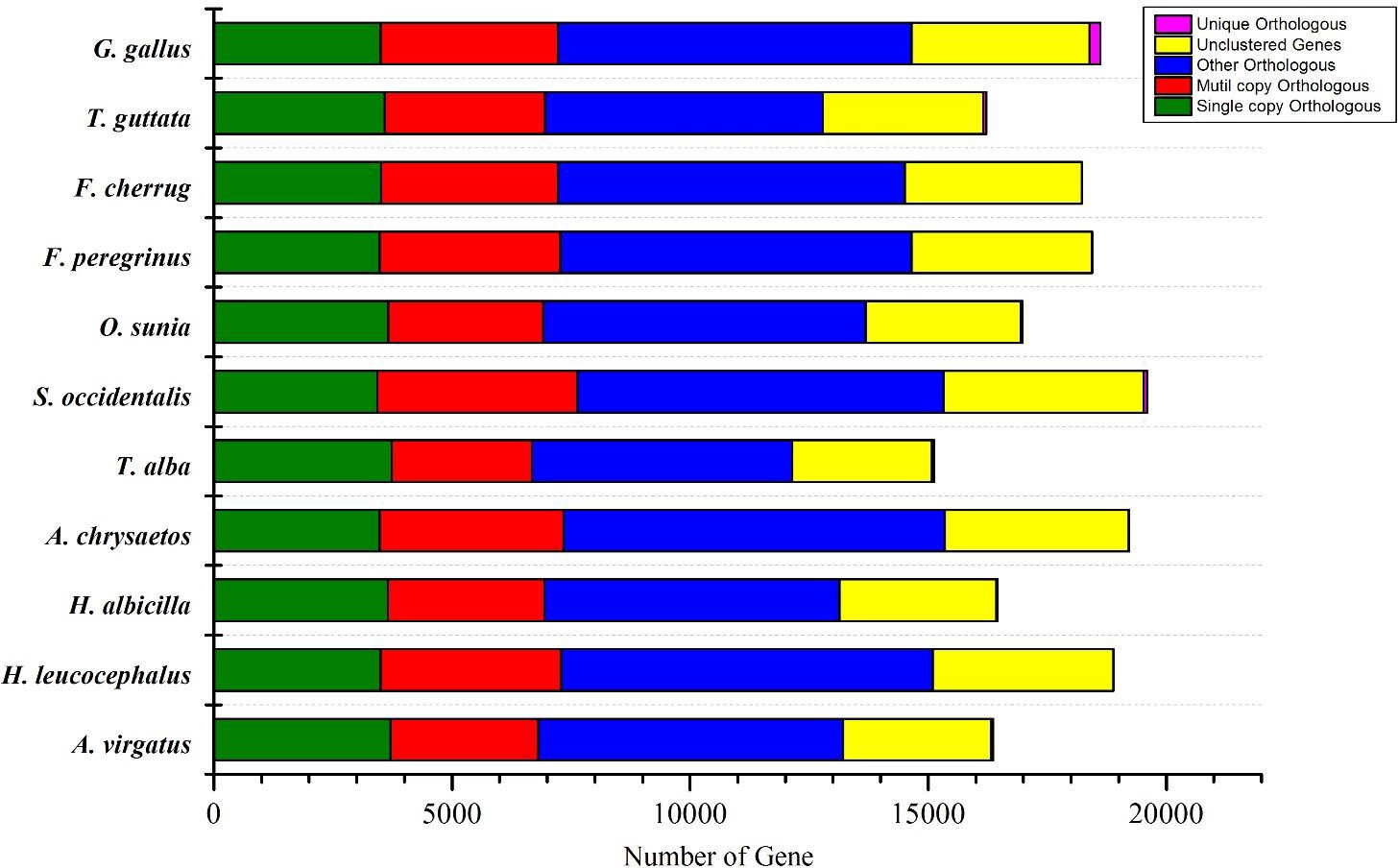


**Supplementary Fig. S8** Orthologous gene families in 13 birds. This figure was drawn based on Supplementary Table S16.

# Supplementary Tables

**Supplementary Table S1 Statistics of clean data for *A. virgatus.***

| Insert  size (bp) | Reads length  (bp) | Reads number | Total bases (Gb) | Physical depth (X) |
| --- | --- | --- | --- | --- |
| 230 | 150 | 431548038 | 64.73 | 54.86 |
| 500 | 150 | 259292274 | 38.89 | 32.96 |
| 2000 | 150 | 177573690 | 26.64 | 22.58 |
| 5000 | 150 | 129801640 | 19.47 | 16.5 |
| 10000 | 150 | 127628194 | 19.14 | 16.22 |
| Total |  | 1125843836 | 168.87 | 143.11 |

The genome size of *A. virgatus* is estimated to be 1.18 Gb (Supplementary Table S3).

**Supplementary Table S2 Statistics of clean data for *O. sunia.***

| Insert size (bp) | Reads length (bp) | Reads number | Total bases (Gb) | Physical depth (X) |
| --- | --- | --- | --- | --- |
| 230 | 150 | 393563172 | 59.03 | 45.41 |
| 500 | 150 | 264164814 | 39.62 | 30.48 |
| 2000 | 150 | 140215754 | 21.03 | 16.18 |
| 5000 | 150 | 124827242 | 18.72 | 14.4 |
| 10000 | 150 | 124288330 | 18.64 | 14.34 |
| Total |  | 1047059312 | 157.04 | 120.8 |

The genome size of *O. sunia* is estimated to be 1.3 Gb.

**Supplementary Table S3 Statistics of 23-mers analyses of *A. virgatus* and *O. sunia* genomes.**

| Species | K number | K depth | Genome size  estimation | Used bases | Used reads | Physical  coverage(X) |
| --- | --- | --- | --- | --- | --- | --- |
| *O. sunia* | 44,093,396,108 | 34 | 1,296,864,591 | 59,034,475,800 | 393,563,172 | 45.5 |
| *A. virgatus* | 45,892,053,130 | 39 | 1,176,719,311 | 64,732,205,700 | 431,548,038 | 55 |

**Supplementary Table S4 Assembly information of 13 bird species.**

| Common name | Species name | Genome  size (Gb) | Scaffold  N50 (Mb) | Reference |
| --- | --- | --- | --- | --- |
| besra | *Accipiter virgatus* | 1.184 | 5.3758 | This study |
| bald eagle | *Haliaeetus leucocephalus* | 1.1784 | 9.1455 | ^1^ |
| white-tailed eagle | *Haliaeetus albicilla* | 1.1335 | 0.0573 | ^1^ |
| golden eagle | *Aquila chrysaetos* | 1.1927 | 9.2307 | ^2^ |
| barn owl | *Tyto alba* | 1.1201 | 0.0528 | ^1^ |
| spotted owl | *Strix occidentalis* | 1.2555 | 3.983 | ^3^ |
| oriental scops owl | *Otus sunia* | 1.2475 | 7.7932 | This study |
| Saker falcon | *Falco cherrug* | 1.1748 | 4.1545 | ^4^ |
| peregrine falcon | *Falco peregrinus* | 1.172 | 3.9358 | ^4^ |
| zebra finch | *Taeniopygia guttata* | 1.2321 | 62.375 | ^5^ |
| chicken | *Gallus gallus* | 1.2303 | 82.3102 | ^6^ |
| chuck-will's-widow | *Antrostomus carolinensis* | 1.1197 | 0.0463 | ^1^ |
| brown kiwi | *Apteryx australis* | 1.524 | 5.679 | ^7^ |

**Supplementary Table S5 Statistics of *A. virgatus* and *O. sunia* assemblies.**

|  | *A. virgatus* |  | *O. sunia* |  |
| --- | --- | --- | --- | --- |
|  | Scaffold |  | Scaffold |  |
|  | Length (bp) | Number | Length (bp) | Number |
| N90 | 899,688 | 267 | 915,890 | 208 |
| N80 | 1,776,807 | 175 | 2,213,404 | 124 |
| N70 | 2,718,730 | 122 | 3,945,905 | 83 |
| N60 | 3,806,162 | 85 | 6,381,188 | 58 |
| N50 | 5,375,830 | 59 | 7,793,190 | 41 |
| N40 | 6,765,049 | 39 | 11,901,972 | 27 |
| N30 | 9,550,654 | 25 | 15,784,118 | 18 |
| N20 | 13,123,630 | 14 | 20,596,581 | 11 |
| N10 | 16,778,946 | 7 | 23,048,032 | 5 |
| Max length | 22,545,549 |  | 31,196,950 |  |
| Total length | 1,183,976,409 |  | 1,247,510,747 |  |
| Number >= 2000 |  | 2,187 |  | 3,870 |

**Supplementary Table S6 Statistics of the genome completeness of *A. virgatus* genome based on 248 CEGs.**

|  | prots | Percentage (%) |
| --- | --- | --- |
| Completeness | 181 | 72.98 |
| Group1 | 49 | 74.24 |
| Group2 | 44 | 78.57 |
| Group3 | 41 | 67.21 |
| Group4 | 47 | 72.31 |
| Partial | 219 | 88.31 |
| Group1 | 58 | 87.88 |
| Group2 | 53 | 94.64 |
| Group3 | 49 | 80.33 |
| Group4 | 59 | 90.77 |

**Supplementary Table S7 Statistics of the genome completeness of *O. sunia* genome based on 248 CEGs.**

|  | prots | Percentage (%) |
| --- | --- | --- |
| Completeness | 208 | 83.87 |
| Group1 | 54 | 81.82 |
| Group2 | 50 | 89.29 |
| Group3 | 49 | 80.33 |
| Group4 | 55 | 84.62 |
| Partial | 229 | 92.34 |
| Group1 | 61 | 92.42 |
| Group2 | 54 | 96.43 |
| Group3 | 55 | 90.16 |
| Group4 | 59 | 90.77 |

**Supplementary Table S8 Statistics of the genome completeness of the genome completeness of *A. virgatus* based on BUSCO benchmark.**

| **BUSCO benchmark** | **Number** | **Percentage (%)** |
| --- | --- | --- |
| **Complete BUSCOs** | 251 | 83 |
| **Complete Single-Copy BUSCOs** | 246 | 81.3 |
|  |  |  |
| **Complete Duplicated BUSCOs** | 5 | 1.7 |
|  |  |  |
| **Fragmented BUSCOs** | 26 | 8.5 |
|  |  |  |
| **Missing BUSCOs** | 26 | 8.5 |
|  |  |  |
| **Total BUSCO groups searched** | 303 | 100 |
|  |  |  |

**Supplementary Table S9 Statistics of the genome completeness of the genome completeness of *O. sunia* based on BUSCO benchmark.**

| **BUSCO benchmark** | **Number** | **Percentage (%)** |
| --- | --- | --- |
| **Complete BUSCOs** | 282 | 93.1 |
| **Complete Single-Copy BUSCOs** | 275 | 90.8 |
|  |  |  |
| **Complete Duplicated BUSCOs** | 7 | 2.3 |
|  |  |  |
| **Fragmented BUSCOs** | 7 | 2.3 |
|  |  |  |
| **Missing BUSCOs** | 14 | 4.6 |
|  |  |  |
| **Total BUSCO groups searched** | 303 | 100 |
|  |  |  |

**Supplementary Table S10 Statistics of repetitive elements in genomes of *A. virgatus* and *O. sunia.***

|  |  | *A. virgatus* |  |  | *O. sunia* |  |
| --- | --- | --- | --- | --- | --- | --- |
| Type | Number | Length (bp) | Percentage (%) | Number | Length (bp) | Percentage (%) |
| DNAa | 53,439 | 6,213,395 | 0.52 | 54,191 | 6,380,385 | 0.51 |
| LINEsb | 95,395 | 35,141,391 | 2.97 | 133,808 | 54,039,277 | 4.33 |
| SINEsc | 12,033 | 1,548,756 | 0.13 | 11,644 | 1,481,285 | 0.12 |
| LTRd | 51,577 | 20,555,613 | 1.74 | 43,153 | 15,090,790 | 1.21 |
| Unclassified | 1,770 | 105,613 | 0.01 | 2,023 | 179,163 | 0.01 |
| Total | 214,214 | 63,564,768 | 5.37 | 244,819 | 77,170,900 | 6.19 |

^a^ DNA: DNA transposons.

^b^ LINE: Long Interspersed Nuclear Elements.

^c^ SINE: Short Interspersed Nuclear Elements.

^d^ LTR: Long Terminal Repeated Elements.

**Supplementary Table S11 Statistics of predicted protein-coding genes for *A. virgatus.***

| **Gene set** | | **Gene Number** | **Transcript number** | **Average transcript length (bp)** | **total number of exon** | **total number of intron** | **Average CDS length (bp)** | **Average exons per gene** | **Average exon length (bp)** | **Average intron length (bp)** |
| --- | --- | --- | --- | --- | --- | --- | --- | --- | --- | --- |
| ***De novo*** | **Augustus** | 18,046 | 18046 | 23106 | 158,457 | 140,411 | 1493.87 | 8.78 | 170.13 | 2777.64 |
|  | **Genscan** | 40,911 | 40911 | 21523.4 | 318,448 | 277,537 | 1340.71 | 7.78 | 172.24 | 2975.08 |
| **Homolog** | **GeneWise** | 16,388 | 16388 | 22236.7 | 143,565 | 127,177 | 1576.86 | 8.76 | 180 | 2662.23 |
| **integrated** | **EVM** | 16,388 | 16388 | 27139.3 | 161,213 | 144,825 | 1664.77 | 9.84 | 169.23 | 2882.63 |

**Supplementary Table S12 Statistics of predicted protein-coding genes for *O. sunia.***

| **Gene set** | | **Gene Number** | **Transcript number** | **Average transcript length (bp)** | **total number of exon** | **total number of intron** | **Average CDS length (bp)** | **Average exons per gene** | **Average exon length (bp)** | **Average intron length (bp)** |
| --- | --- | --- | --- | --- | --- | --- | --- | --- | --- | --- |
| ***De novo*** | **Augustus** | 20,863 | 20,863 | 20920.9 | 176,409 | 155,546 | 1449.87 | 8.46 | 171.47 | 2611.6 |
|  | **Genscan** | 40,341 | 40,341 | 22468.1 | 318,090 | 277,749 | 1330.59 | 7.89 | 168.75 | 3070.07 |
| **Homolog** | **GeneWise** | 18,332 | 18,332 | 21132.8 | 154,699 | 136,367 | 1504.5 | 8.44 | 178.28 | 2638.66 |
| **Integrated** | **EVM** | 15,229 | 15,229 | 29744.2 | 170,635 | 155,406 | 1850.52 | 11.2 | 165.16 | 2733.44 |

**Supplementary Table S13 Functional annotation of *A. virgatus* and *O. sunia* genomes.**

| *A. virgatus* | | | | *O. sunia* | |
| --- | --- | --- | --- | --- | --- |
|  |  | Number | Percentage (%) | Number | Percentage (%) |
|  | SwissProt | 13661 | 83.36 | 14051 | 92.26 |
|  | TrEMBL | 14175 | 86.5 | 14338 | 94.15 |
|  | KEGG | 8864 | 54.09 | 9355 | 61.43 |
| Annotated | InterPro | 13894 | 84.78 | 14145 | 92.88 |
|  | GO | 13557 | 89.02 | 14222 | 86.78 |
|  | Nr | 14222 | 86.78 | 14340 | 94.16 |
|  | all | 14398 | 87.86 | 14416 | 94.66 |
| Unannotated |  | 1990 | 12.14 | 813 | 5.34 |
| Total |  | 16388 |  | 15229 |  |

**Supplementary Table S14 Summary of the ncRNA in *A. virgatus.***

| Type |  | Copy | Average length  (bp) | Total length (bp) |
| --- | --- | --- | --- | --- |
| miRNA |  | 165 | 84.8 | 13,992 |
| tRNA |  | 200 | 74.5 | 14,899 |
| 5S rRNA |  | 55 | 79.2 | 4,355 |
|  | CD-box | 89 | 91 | 8,100 |
| snRNA | HACA-box | 55 | 139 | 7,646 |
|  | splicing | 9 | 140.3 | 1,263 |

**Supplementary Table S15 Summary of the ncRNA in *O. sunia.***

| Type |  | Copy | Average length (bp) | Total length (bp) |
| --- | --- | --- | --- | --- |
| miRNA |  | 212 | 85 | 18,016 |
| tRNA |  | 281 | 75.8 | 21,290 |
| 5S rRNA |  | 53 | 88.7 | 4,701 |
|  | CD-box | 112 | 96.4 | 10,794 |
| snRNA | HACA-box | 65 | 142.2 | 9,242 |
|  | splicing | 30 | 128.8 | 3,864 |

**Supplementary Table S16 Summary of orthologous gene families of thirteen birds.**

| Species name | Single copy orthologousa | Mutil copy orthologousb | Unclustered genesc | Unique orthologousd | Other orthologouse |
| --- | --- | --- | --- | --- | --- |
| *A. virgatus* | 3,712 | 3,098 | 3,120 | 52 | 6,406 |
| *H. leucocephalus* | 3,500 | 3,795 | 119 | 0 | 7,798 |
| *H. albicilla* | 3,653 | 3,292 | 1,734 | 23 | 6,193 |
| *A. chrysaetos* | 3,484 | 3,854 | 270 | 12 | 8,017 |
| *T. alba* | 3,740 | 2,939 | 1,730 | 43 | 5,463 |
| *S. occidentalis* | 3,433 | 4,200 | 1,323 | 71 | 7,691 |
| *O. sunia* | 3,657 | 3,256 | 1,512 | 27 | 6,777 |
| *F. peregrinus* | 3,483 | 3,786 | 189 | 6 | 7,383 |
| *F. cherrug* | 3,506 | 3,717 | 186 | 0 | 7,286 |
| *T. guttata* | 3,585 | 3,364 | 767 | 66 | 5,842 |
| *G. gallus* | 3,499 | 3,729 | 689 | 227 | 7,425 |
| *A. carolinensis* | 3,608 | 3,353 | 2,260 | 43 | 6,187 |
| *A. australis* | 3,476 | 3,872 | 1,483 | 99 | 7,321 |

a Single copy orthologous include the common orthologous with the same number of copies in different species.

b Mutil copy orthologous include the common orthologous with different copy numbers in the different species.

c Unclustered genes include the genes that cannot be clustered into known gene families.

d Unique orthologous include the orthologous just in one species.

e Other orthologous include the genes that can be clustered into known gene families, but it not belongs to other categories.

We identified 2,845 1:1:1 orthologous gene in all species.

**Supplementary Table S17 PSGs in accipitrid and owl branches.**

|  | **Accipitrids** | **Owls** |
| --- | --- | --- |
| **1** | ***SLC15A5*** | ***ACOX3*** |
| **2** | ***FAM114A2*** | ***KBTBD12*** |
| **3** | **novel gene** | ***FHL1*** |
| **4** | **novel gene** | ***SDR16C5*** |
| **5** | ***DTHD1*** | **novel gene** |
| **6** | ***SLC2A12*** | ***DTHD1*** |
| **7** | ***ACE2*** | ***LETM1*** |
| **8** | ***SLC16A4*** | ***RASEF*** |
| **9** | ***YAE1*** | **novel gene** |
| **10** | ***UMODL1*** | ***COL3A1*** |
| **11** | ***ATXN7L1*** | ***STOX2*** |
| **12** | ***COL3A1*** | ***USP16*** |
| **13** | ***IL17RD*** | ***ECI2*** |
| **14** | **novel gene** | ***STX8*** |
| **15** | ***CDK15*** | ***HAUS8*** |
| **16** | ***TXNDC5*** | ***DDX24*** |
| **17** | ***TMPO*** | ***EPM2A*** |
| **18** | ***WASHC5*** | ***TICAM1*** |
| **19** | ***STN1*** | ***RPL6*** |
| **20** | ***TOMM70*** | ***PPP4R4*** |
| **21** | ***UPK1B*** | ***SYNPO2L*** |
| **22** | ***ABCB7*** | ***WDR3*** |
| **23** | ***VEPH1*** | ***ABRA*** |
| **24** | ***USP1*** | **novel gene** |
| **25** | ***CEP104*** | ***WDR24*** |
| **26** | ***IMPA2*** | ***TEX11*** |
| **27** | ***POLR1E*** | **novel gene** |
| **28** | ***PCTP*** | ***QTRT2*** |
| **29** | ***MDM2*** | ***TSPAN8*** |
| **30** | ***HAUS8*** | ***RHAG*** |
| **31** | ***RWDD1*** | ***IL13RA1*** |
| **32** | **novel gene** | ***ARHGAP20*** |
| **33** | ***ARV1*** | ***TAF1D*** |
| **34** | ***AMD1*** | ***ZWILCH*** |
| **35** | ***AMN1*** | ***SYT16*** |
| **36** | **novel gene** | ***TMEM150C*** |
| **37** | ***ZP2*** | ***FASTKD1*** |
| **38** | ***ASB3*** | **novel gene** |
| **39** | ***ABRA*** | ***HPF1*** |
| **40** | ***CAPS2*** | ***PLA2G7*** |
| **41** | ***CD74*** | ***TGFBR3*** |
| **42** | **novel gene** | ***TEKT4*** |
| **43** | ***GIPC2*** | ***FCF1*** |
| **44** | ***EFCAB7*** | ***ARHGEF12*** |
| **45** | ***CEP57L1*** | ***TNNC1*** |
| **46** | ***LIMCH1*** | **novel gene** |
| **47** | ***HEMK1*** | ***ALCAM*** |
| **48** | ***EDEM1*** | **novel gene** |
| **49** | ***SSPN*** | ***SCP2*** |
| **50** | ***RHAG*** | ***NDUFAF1*** |
| **51** | **novel gene** | ***VRK1*** |
| **52** | ***NAGA*** | ***CFI*** |
| **53** | **novel gene** | **novel gene** |
| **54** | ***IDNK*** | ***ALPK1*** |
| **55** | ***NUB1*** | ***TICRR*** |
| **56** | ***ZNF277*** | **novel gene** |
| **57** | ***LRRN4*** | ***POR*** |
| **58** | ***CENPF*** | ***IFNAR2*** |
| **59** | ***CD44*** | **novel gene** |
| **60** | ***OGFOD2*** | ***DRC3*** |
| **61** | ***TAF1D*** | ***HSF3*** |
| **62** | ***YARS*** |  |
| **63** | ***DCP2*** |  |
| **64** | **novel gene** |  |
| **65** | ***TSPAN9*** |  |
| **66** | **novel gene** |  |
| **67** | ***PSMB2*** |  |
| **68** | ***CTNS*** |  |
| **69** | ***RHCE*** |  |
| **70** | ***MOSPD1*** |  |
| **71** | ***MAP3K14*** |  |
| **72** | ***PRMT9*** |  |
| **73** | ***PWP1*** |  |
| **74** | **novel gene** |  |
| **75** | **novel gene** |  |
| **76** | ***RPL9*** |  |
| **77** | ***PUS7L*** |  |
| **78** | ***PRIMPOL*** |  |
| **79** | ***THADA*** |  |
| **80** | ***XRCC5*** |  |
| **81** | **novel gene** |  |
| **82** | **novel gene** |  |
| **83** | ***SPP2*** |  |
| **84** | **novel gene** |  |
| **85** | ***KIF4A*** |  |
| **86** | ***IFNG*** |  |
| **87** | ***PLPP4*** |  |
| **88** | ***COQ2*** |  |
| **89** | ***PDSS1*** |  |
| **90** | ***KLHL34*** |  |
| **91** | ***ZNF839*** |  |
| **92** | ***STYK1*** |  |
| **93** | ***NOC4L*** |  |
| **94** | ***TMEM181*** |  |
| **95** | ***RHBG*** |  |
| **96** | ***PYROXD1*** |  |
| **97** | **novel gene** |  |
| **98** | ***HSPB3*** |  |
| **99** | ***FAM120B*** |  |
| **100** | ***HNRNPH2*** |  |
| **101** | ***LRRC34*** |  |
| **102** | ***PNLDC1*** |  |
| **103** | ***TICRR*** |  |
| **104** | ***MCFD2*** |  |
| **105** | ***SIRT1*** |  |
| **106** | ***GGT5*** |  |
| **107** | ***THAP12*** |  |
| **108** | ***USP8*** |  |
| **109** | ***CEP63*** |  |
| **110** | ***TPP2*** |  |
| **111** | ***VSIG1*** |  |
| **112** | ***METTL21C*** |  |
| **113** | ***C1GALT1*** |  |
| **114** | ***ELMOD1*** |  |
| **115** | ***SLC7A6OS*** |  |
| **116** | ***SIRT6*** |  |
| **117** | **novel gene** |  |
| **118** | ***SLBP*** |  |

**Supplementary Table S18 Annotation of olfactory receptor genes in thirteen birds.**

| Species | Intact | Other | Total |
| --- | --- | --- | --- |
| *A. virgatus* | 41 | 53 | 94 |
| *A. carolinensis* | 39 | 62 | 101 |
| *A. australis* | 43 | 61 | 104 |
| *A. chrysaetos* | 39 | 71 | 110 |
| *F. cherrug* | 23 | 60 | 83 |
| *F. peregrinus* | 24 | 55 | 79 |
| *G. gallus* | 29 | 93 | 122 |
| *H. albicilla* | 31 | 61 | 92 |
| *H. leucocephalus* | 33 | 70 | 103 |
| *O. sunia* | 46 | 55 | 101 |
| *S. occidentalis* | 45 | 64 | 109 |
| *T. guttata* | 11 | 81 | 92 |
| *T. alba* | 29 | 65 | 94 |

The number of ORs in this study has some difference with previous studies^4,7^, which possibly caused by the difference of methods and OR reference database.

**Supplementary Table S19 The genome information of thirteen species used in this study.**

| Order | Family | Common name | Species name | From | Assembly ID |
| --- | --- | --- | --- | --- | --- |
|  |  | Besra | *Accipiter virgatus* (*A. virgatus*) | this study |  |
| Accipitriformes | Accipitridae | Bald eagle | *Haliaeetus leucocephalus* (*H. leucocephalus*) | Genbank | Haliaeetus_leucocephalus-4.0 |
|  |  | White-talied eagle | *Haliaeetus albicilla* (*H. albicilla*) | Genbank | ASM69140v1 |
|  |  | Golden eagle | *Aquila chrysaetos* (*A. chrysaetos*) | Genbank | Aquila_chrysaetos-1.0.2 |
|  | Tytonidae | Barn owl | *Tyto alba* (*T. alba*) | Genbank | ASM68720v1 |
| Strigiformes | Strigidae | Northern spotted owl | *Strix occidentalis* (*S. occidentalis*) | Genbank | Soccid_v01 |
|  |  | Oriental scops owl | *Otus sunia* (*O. sunia*) | this study |  |
| Falconiformes | Falconidae | Peregrine falcon | *Falco peregrinus* (*F. peregrinus*) | Genbank | F_peregrinus_v1.0 |
|  |  | Saker falcon | *Falco cherrug* (*F. cherrug*) | Genbank | F_cherrug_v1.0 |
| Passeriformes | Estrildidae | Zebra finch | *Taeniopygia guttata* (*T. guttata*) | Genbank | Taeniopygia_guttata-3.2.4 |
| Galliformes | Phasianidae | Red junglefowl | *Gallus gallus* (*G. gallus*) | Genbank | Gallus_gallus-5.0 |
| Caprimulgiformes | Caprimulgidae | Chuck-will's-widow | *Antrostomus carolinensis* (*A. carolinensis*) | Genbank | ASM70074v1 |
| Apterygiformes | Apterygidae | Brown kiwi | *Apteryx australis* (*A. australis*) | Genbank | AptMant0 |

**Supplementary Table S20 Owl samples for PCR validation of owl-specific missense mutations in *ALCAM*.**

| Sample | Order | Family | Common name | Species name |
| --- | --- | --- | --- | --- |
| owls-1 | Strigiformes | Tytonidae | Eastern grass owl | *Tyto longimembris* |
| owls-2 |  |  |  |  |
| owls-3 |  |  |  |  |
| owls-4 |  |  |  |  |
| owls-5 |  |  |  |  |
| owls-6 |  |  |  |  |
| owls-7 |  |  |  |  |
| owls-8 |  |  |  |  |
| owls-9 |  | Strigidae | Short-eared owl | *Asio flammeus* |
| owls-10 |  |  |  |  |
| owls-11 |  |  | Long-eared owl | *Asio otus* |
| owls-12 |  |  |  |  |
| owls-13 |  |  | Asian barred owlet | *Glaucidium cuculoides* |
| owls-14 |  |  | Brown wood owl | *Strix leptogrammica* |
| owls-15 |  |  | Oriental scops owl | *Otus sunia* |
| owls-16 |  |  |  |  |
| owls-17 |  |  | Indian scops owl | *Otus bakkamoena* |
| owls-18 |  |  | Eurasian eagle-owl | *Bubo bubo* |
| owls-19 |  |  | Little owl | *Athene noctua* |
| owls-20 |  |  | Collared owlet | *Glaucidium brodiei* |

**Supplementary Table S21 Primers used to amplify the mutation sites in *ALCAM*.**

| **Primer name** | **Primer sequence (5'-3')** | | **Reference** |
| --- | --- | --- | --- |
|  | **Forward** | **Reverse** |  |
| ALCAM_M | TTCAGAACAACCACATCA | GTAATGTTTTTAGCAGGC | This study |
| ALCAM_L | GCAGGACTTACTGGAATG | GCGTAGTGTTACTGTGTG | This study |

**Supplementary Table S22 Dietary information of 30 birds.**

| Species name | Order | Fimily | Diet |
| --- | --- | --- | --- |
| *Accipiter virgatus* | Accipitriformes | Accipitridae | Carnivore |
| *Aquila chrysaetos* | Accipitriformes | Accipitridae | Carnivore |
| *Falco cherrug* | Falconiformes | Falconidae | Carnivore |
| *Falco peregrinus* | Falconiformes | Falconidae | Carnivore |
| *Otus sunia* | Strigiformes | Strigidae | Carnivore |
| *Strix occidentalis* | Strigiformes | Strigidae | Carnivore |
| *Tyto alba* | Strigiformes | Tytonidae | Carnivore |
| *Ciconia boyciana* | Ciconiiformes | Ciconiidae | Carnivore |
| *Egretta garzetta* | Pelecaniformes | Ardeidae | Carnivore |
| *Phaethon lepturus* | Phaethontiformes | Phaethontidae | Carnivore |
| *Phalacrocorax brasilianus* | Pelecaniformes | Phalacrocoracidae | Carnivore |
| *Phoenicopterus ruber* | Phoenicopteriformes | Phoenicopteridae | Carnivore |
| *Apteryx australis* | Apterygiformes | Apterygidae | Carnivore |
| *Arborophila ardens* | Galliformes | Phasianidae | Omnivore |
| *Arborophila rufipectus* | Galliformes | Phasianidae | Omnivore |
| *Meleagris gallopavo* | Galliformes | Phasianidae | Omnivore |
| *Coturnix japonica* | Galliformes | Phasianidae | Omnivore |
| *Gallus gallus* | Galliformes | Phasianidae | Omnivore |
| *Numida meleagris* | Galliformes | Numididae | Omnivore |
| *Balearica regulorum* | Gruiformes | Gruidae | Omnivore |
| *Picoides pubescens* | Piciformes | Picidae | Omnivore |
| *Parus major* | Passeriformes | Paridae | Omnivore |
| *Passer domesticus* | Passeriformes | Passeridae | Omnivore |
| *Serinus canaria* | Passeriformes | Fringillidae | Herbivore |
| *Taeniopygia guttata* | Passeriformes | Estrildidae | Herbivore |
| *Geospiza fortis* | Passeriformes | Thraupidae | Herbivore |
| *Lepidothrix coronata* | Passeriformes | Pipridae | Herbivore |
| *Lonchura striata* | Passeriformes | Estrildidae | Herbivore |
| *Colinus virginianus* | Galliformes | Odontophoridae | Herbivore |
| *Anser cygnoides* | Anseriformes | Anatidae | Herbivore |

The diet information is available at HBW(www.hbw.com).

# Supplementary Note

# Construction of Genomic Libraries, Sequencing, and Filtering

# To assemble the genomes of *A. virgatus* and *O. sunia*, we generated both short-insert-size libraries (250 bp and 500 bp) and long-insert-size libraries (2 kb, 5 kb, and 10 kb) using genomic DNA of *A. virgatus* and *O. sunia*. In this study, Illumina HiSeq 2000 sequencing platform was employed after library construction and quality control.

# There were mis-sequencing and unqualified reads and reads with adapter in the raw sequencing data. In order to reduce the adverse effects of the bad reads on the genome assembly, it is necessary to check and filter the raw data generated by Illumina HiSeq 2000 to get clean data. We filtered out the bad reads as follows:

# 1) Reads with adapter sequence contamination;

# 2) Reads with > 5% ambiguous bases;

# 3) Reads with low-quality bases (≥ 20% bases with Illumina Q-value ≤ 5 for the reads);

# 4) duplication (reads1 and reads 2 were the same);

# 5) The two reads overlapped 10 bp and the mismatch was < 10%.

# In sum, approximately 169 Gb and 157 Gb clean data were obtained for *A. virgatus* (Table S1) and *O. sunia* (Table S2), respectively after filtering out the bad reads. The clean data were used for following de novo assembly and analyses.

# Genome Size Estimation

# K-mer analysis has previously been used to estimate genome size^8^. A K-mer refers to an artificial sequence division of k nucleotides. The genome sequencing read with L base pairs(bp) contains L-k+1 K-mers if each K-mer's length is k bp. The frequency of each K-mer can be calculated from the sequencing read data. The frequency of each K-mer follows a Poisson distribution in a given data set except for a high proportion of low frequency K-mers due to sequencing errors^9^. Thus, the genome size (G) is calculated as G = k_num / k_depth (k_num is the total number of K-mer, k_depth is the maximal frequency). In our study, we performed K-mer analysis by short insert size library reads to estimate genome size of *A. virgatus* and *O. sunia* based on k=23. We used 59.03 Gb (*O. sunia*) and 64.73 Gb (*A. virgatus*) sequencing data to estimate genome size. The frequencies of all 23-mers in the sequence data were counted using Jellyfish^10^. The frequency distribution of the 23-mers derived from the sequence read data is plotted in Fig. S1 and Fig. S2 for A. virgatus and *O. sunia*, respectively. The results of 23-mers analyses of *A. virgatus* and *O. sunia* genomes were shown in Table S3. In the end, the genome size of *A. virgatus* and *O. sunia* was estimated to be 1.30 Gb and 1.18 Gb, respectively. And we found no significant repeat content and prominent heterozygosity in each genome.

# Genome Assembly

# After quality control, total of 168.87 Gb and 157.04 Gb clean data of A. virgatus and *O. sunia* were obtained and used for de novo genome assembly by Soapdenovo2^11^, respectively. In these processes, short-insert-size libraries were used in both contig and scaffold construction, and long-insert-size libraries were only used in scaffold construction. The parameters used for *A. virgatus* genome assembly were set as k=27 and M=2, whereas k=33 and M=2 for *O. sunia* SSPACE^12^ was used to build super-scaffolds. SOAP Gapcloser was used to close the gap in the scaffold of each genome with default parameters by the corrected short insert libraries.

# In the end, the assembled length of *A. virgatus* and *O. sunia* was 1.18 Gb with scaffold N50 5.38 Mb, and 1.25 Gb with scaffold N50 7.79 Mb, respectively (Table S5). All subsequent analyses were done based on the gap-closed assembles.

# Genome Completeness Assessment

# Firstly, comparison of the GC content of 13 genomes including *A. virgatus* and *O. sunia* was conducted to analyze nucleotide distribution (Fig. S3). The results showed that the GC content in both genomes was distributed in a relatively concentrated region.

# CEGMA (Version 2.5)^13^ and BUSCO (Version)^14^ have also been used to assess genome assemblies. The Core Eukaryotic Genes Mapping Approach (CEGMA) was a classical approach used to check for the existence of core eukaryotic genes in genome assemblies. Default parameters except --vrt were used in CEGMA, and results showed that 72.98% and 83.87% complete gene sets could be found in the final assembled genomes of *A. virgatus* (Table S6) and *O. sunia* (Table S7), respectively. Bench marking Universal Single-Copy Orthologs (BUSCO) was a new approach to assess genome completeness on the basis of evolutionarily informed expectations of gene content. With default parameters, BUSCO results showed that 83% and 93.1% of the eukaryotic single-copy genes were captured in the final assembled genomes of *A. virgatus* (Table S8) and *O. sunia* (Table S9), respectively.

# Repeat Annotation

# We performed Repbase-based approaches to identify repeat sequences in nine raptor genomes including the genomes assembled in this study (Fig. S4), and compared the average percentage of each repeat element in all raptor genomes (Fig. S5).

# Firstly, we matched to entries in a custom library for known repetitive sequences using RepeatMasker (Version4.0.5)^15^ with appropriate default parameters to find repeats. Then, based on the annotation results of the repeat sequences, we counted the repeat elements (Table S10) and masked the repetition of the genomes sequenced in this study (the nucleotide base was replaced by N) for the de novo gene prediction.

# Gene Prediction

# We combined homology-based and de novo methods to predict protein-coding genes in both genomes. For de novo prediction, Augustus (Version 3.2.1)^16^ and Genscan (Version 1.0)^17^ were used to predict protein-coding genes in the repeat-masked genomes. For Augustus, we used 15,737 genes from Falco cherrug genome to train gene model parameters. Using this software, we identified 18,046 and 20,863 genes in *A. virgatus* (Table S11) and *O. sunia* (Table S12), respectively. The parameters for Genscan were derived from human studies^4^, and we identified 40,911 and 40,341 genes in *A. virgatus* (Table S11) and *O. sunia* (Table S12), respectively.

# For homology-based prediction, homologous protein sequences of four birds were downloaded from public genome databases (Gallus gallus and Homo spiens from Ensembl- 82, and Falco cherrug and Falco peregrinus from Genbank). All the protein sequences were mapped to the *A. virgatus* and *O. sunia* genomes using TBLASTN (Version2.2.28+)^18^ (E-value cutoff 1e-5). We removed the sequences that matches with length coverage <25% of the homologous proteins, others were considered as gene model candidates and identified by software SOLAR^19^. Finally, the gene structure was further identified by GeneWise (Version 2.4.1)^20^. We identified16,388 and 18,332 in *A. virgatus* (Table S11) and *O. sunia* (Table S12), respectively.

# Finally, the results generated above were integrated using EVM^21^ to form a comprehensive and non-redundant reference gene sets. In the end, 16,388 and 15,229 gens were identified in *A. virgatus* (Table S11) and *O. sunia* (Table S12), respectively.

# Non-coding RAN (ncRNA) Prediction

# The tRNA genes were identified by tRNAscan-SE (Version 1.3.1)^22^ with parameters used - A -B and -O. For 5S rRNA identification, we downloaded the avian 5S rRNA sequences from the Ensembl and Genbank database. Then rRNAs in the database were aligned against the *A. virgatus* and *O. sunia* genomes using blastn with cutoff of E-value <1e-5, identity ≥85%. Based on the results of blastn, we got the result of rRNA annotation by removing repetitive regions. We used INFERNAL (Version 1.0.2)^23^ by searching against the Rfam^24^ database with default parameters to identify the other ncRNAs, including miRNA and snRNA. The distribution of ncRNA in *A. virgatus* and *O. sunia* genomes was shown in Table S14 and Table S15, respectively.

# Orthologous Gene Family Clustering

# Genome sequences and coding annotation files for 11 species mention in Table S16 were downloaded from public genome databases. We used these eleven species, together with two genomes we assembled, to cluster orthologous gene family. Firstly, we extracted the longest coding sequences of each gene according to the annotation files. The genes with less than ten amino acids were removed. Then we used OrthoMCL pipeline (Version 2.0.9)^25^ to cluster gene family with default parameters except –I=1.5, and all- against-all protein searches were performed using blastp with e-value cutoff at 1e-5. A total of 16,530 orthologous gene families were identified, and 2,845 1:1:1 orthologous gene families shared in all thirteen species (Fig. S8 and Table S16).

# Phylogenetic Tree Construction

# To construct the phylogenetic tree for 13 species mentioned above, a total 2,845 1:1:1 orthologous genes clustered by OrthoMCL have been used. 1) The coding sequences of these genes were aligned using PRANK^26^ respectively. 2) The aligned coding sequences of each species were concatenated by MEGA7^27^. 3) The phylip format of the concatenated coding sequences was obtained using Trimal (Version). 4) The best substitution model was estimated in modeltest 3.7. 4) Phylogenetic tree was constructed by RAxML (Version 8.2.8)^28^. The phylogenetic tree was drawn in FigTree 1.4 (http://tree.bio.ed.ac.uk/software/figtree/) (Fig. 1 in main text)

# Positive Selection Analyses

# A total of 2,845 high-confidence 1:1:1 orthologous genes shared in thirteen species were identified in orthologous gene families clustered. We collected coding sequences of each gene family to detect the positively selected genes. Those multiple sequences were aligned using PRANK. Then aligned sequences were trimmed with default parameters expect –automated which was set to remove potentially unreliably aligned regions and gaps. The detailed information of the positive selection analyses^29^ was shown in the manuscript.

# Analyses of Olfactory Receptor Genes (ORs)

# Reference OR protein sequences were obtained from uniprot (http://www.uniprot.org/) with "Olfactory Receptor" as the key searching word. We combined the ORs and removed the redundant sequences. The reference OR protein sequences were used to blast each of thirteen studied genomes (shown in Fig. 1a in main text) using TBLASTN with E-value = 1E-20. We collected the sequences for putative OR genes, and blast-hits with lowest E-value were retained for the following analysis.

# 1) SOLAR was used to combine fragment sequences into one predicted gene if they belonged to the same query protein.

# 2) Redundant sequences and sequences which were less than 250 bp were removed;

# 3) According to the genomic position, the hits were extracted from each genome. We predicted the gene structure by GeneWise2.2.0.

# 4) The sequences with an associated gene structure were extended by 1.5 kb at both ends, then we checked its open reading frame.

# 5) The genes with a 7TM (Transmembrane) structure (checked using TMHMM2.0^30^), both start codon and stop codon, and no premature stop codon and/or frameshift were identified as intact candidates.

# 6) The final dataset of OR sequences were employed to conduct phylogenetic analysis using MEGA7 for NJ tree. We removed the non-ORs clustered separately from ORs with a high confidence value. The remained genes were defined as intact olfactory receptor genes.

# Analyses of *TAS2R* Genes

# Reference *TAS2R* protein sequences were obtained from uniprot (http://www.uniprot.org/) with "TAS2R" as the key searching word. We combined the *TAS2R*s and removed the redundant sequences. The reference *TAS2R* protein sequences were used to blast each of thirty studied genomes (shown in Fig. 6a in main text) using TBLASTN with E-value = 1E-20. We collected the sequences for putative *TAS2R* genes, and blast-hits with lowest E-value were retained for the following analysis.

# 1) SOLAR was used to combine fragment sequences into one predicted gene if they belonged to the same query protein.

# 2) Redundant sequences and sequences which were less than 300 bp were removed;

# 3) According to the genomic position, the hits were extracted from each genome. We predicted the gene structure by GeneWise2.2.0.

# 4) The sequences with an associated gene structure were extended by 1.5 kb at both ends, then we checked its open reading frame.

# 5) if one sequence possessed at least one premature stop codon and/or frameshift, we categorized it into pseudogene candidates.

# 6) If one sequence had no start or stop codon, we categorized it into partial gene candidates.

# 7) The remaining genes were further checked using TMHMM2. We categorized those sequences with a 7TM (Transmembrane) structure into intact candidates and those without a 7TM treated as a further pseudogene candidate.

# 8) The final dataset of *TAS2R* sequences were employed to conduct phylogenetic analysis using MEGA7 for NJ tree. We removed the non-*TAS2R*s clustered separately from *TAS2R*s with a high confidence value. The remained genes were defined as intact *TAS2R* genes.

# PCR Amplification

# We collected 20 muscle samples of owls (Supplementary Table S20) from the Museum of Sichuan University to validate the owl-specific missense mutation sites in gene *ALCAM*. Both mutation sites were amplified with primers designed by comparing gene sequences of *G.gallus*, *T. guttata*, *A. virgatus*, *F. peregrinus*, and *O. sunia* with Primer Premier 5^31^.

# Primers were 18–23 bp in length and ~50% GC. The primers used in this study were presented in Table S20. TaKaRa RTaq (TaKaRa Biomedical, Japan) was used for the amplification of both mutation sites, and the PCR amplification was performed on a PTC-100 thermal cycler (BioRad, Hercules, CA). The reaction mixture has a total volume of 25μL including 0.2μl ExTaq polymerase (5 U/ml), 1 μl of each primer (10 μM), 1.0~3.0 μl dNTP (2.5 mM each), 2.5 μl of 10x Taq buffer, and about 200 ng total genomic DNA.

# The PCR protocol was as follows: an initial pre-denaturation for 5 min at 94 °C followed by 35 cycles at 94 °C for 30s, 55 °C for 30s, 72 °C for 15s with a final extension at 72 °C for 10 min. The PCR products were electrophoresed on a 1% agarose gel, and then sequenced on an ABI PRISM 3730 DNA sequencer in Tsingke Biotechnology Company (Chengdu, Sichuan Province, China).

# References

# 1 Zhang, G. et al. Comparative genomics reveals insights into avian genome evolution and adaptation. Science 346, 1311-1320 (2014).

# 2 Doyle, J. M. et al. The genome sequence of a widespread apex predator, the golden eagle (Aquila chrysaetos). PloS one 9, e95599 (2014).

# 3 Hanna, Z. R. et al. Northern Spotted Owl (Strix occidentalis caurina) Genome: Divergence with the Barred Owl (Strix varia) and Characterization of Light-Associated Genes. Genome Biology & Evolution 9, 2522 (2017).

# 4 Zhan, X. et al. Peregrine and saker falcon genome sequences provide insights into evolution of a predatory lifestyle. Nature Genetics 45, 563-566 (2013).

# 5 Warren, W. C. et al. The genome of a songbird. Nature 464, 757 (2010).

# 6 Warren, W. C. et al. A New Chicken Genome Assembly Provides Insight into Avian Genome Structure. G3 Genesgenetics 7, 109-117 (2017).

# 7 Duc, D. L. et al. Kiwi genome provides insights into evolution of a nocturnal lifestyle. Genome Biology 16, 147 (2015).

# 8 Z, L. et al. Comparison of the two major classes of assembly algorithms: overlap-layout-consensus and de-bruijn-graph. Briefings in Functional Genomics 11, 25-37 (2012).

# 9 Zeng, X. et al. The draft genome of Tibetan hulless barley reveals adaptive patterns to the high stressful Tibetan Plateau. Proc Natl Acad Sci U S A 112, 1095 (2015).

# 10 Marçais, G. & Kingsford, C. A fast, lock-free approach for efficient parallel counting of occurrences of k-mers. Bioinformatics 27, 764 (2011).

# 11 Luo, R. et al. SOAPdenovo2: an empirically improved memory-efficient short-read de novo assembler. Gigascience 1, 18 (2012).

# 12 Boetzer, M., Henkel, C. V., Jansen, H. J., Butler, D. & Pirovano, W. Scaffolding pre-assembled contigs using SSPACE. Bioinformatics 27, 578-579 (2011).

# 13 Parra, G., Bradnam, K. & Korf, I. CEGMA: a pipeline to accurately annotate core genes in eukaryotic genomes. Bioinformatics 23, 1061 (2007).

# 14 Simão, F. A., Waterhouse, R. M., Ioannidis, P., Kriventseva, E. V. & Zdobnov, E. M. BUSCO: assessing genome assembly and annotation completeness with single-copy orthologs. Bioinformatics 31, 3210-3212 (2015).

# 15 Smit AFA, Hubley R, Green P. 2016. RepeatMasker website and server[CP/OL]. (2016- 9-12)[2016-10-15]. http://www.repeatmasker.org/.

# 16 Stanke, M. et al. AUGUSTUS: ab initio prediction of alternative transcripts. Nucleic Acids Research 34, W435 (2006).

# 17 Burge, C. & Karlin, S. Prediction of complete gene structures in human genomic DNA. Journal of Molecular Biology 268, 78-94 (1997).

# 18 Kent, W. J. BLAT--the BLAST-like alignment tool. Genome research 12, 656-664 (2002).

# 19 Ashburner, M. et al. Gene Ontology: tool for the unification of biology. Nature Genetics 25, 25-29 (2000).

# 20 Birney, E., Clamp, M. & Durbin, R. GeneWise and genomewise. Genome research 14, 988-995 (2004).

# 21 Haas, B. J. et al. Automated eukaryotic gene structure annotation using EVidenceModeler and the Program to Assemble Spliced Alignments. Genome Biology 9, R7 (2008).

# 22 Lowe, T. M. & Eddy, S. R. tRNAscan-SE: a program for improved detection of transfer RNA genes in genomic sequence. Nucleic Acids Research 25, 955-964 (1997).

# 23 Nawrocki, E. P., Kolbe, D. L. & Eddy, S. R. Infernal 1.0: inference of RNA alignments. Bioinformatics 25, 1335 (2009).

# 24 Nawrocki, E. P. et al. Rfam 12.0: updates to the RNA families database. Nucleic Acids Research 43, D130 (2015).

# 25 Li, L., Stoeckert, C. J. & Roos, D. S. OrthoMCL: identification of ortholog groups for eukaryotic genomes. Genome research 13, 2178 (2003).

# 26 Löytynoja, A. & Goldman, N. Phylogeny-aware gap placement prevents errors in sequence alignment and evolutionary analysis. Science 320, 1632-1635 (2008).

# 27 Kumar, S., Stecher, G. & Tamura, K. MEGA7: Molecular Evolutionary Genetics Analysis Version 7.0 for Bigger Datasets. Molecular Biology & Evolution 33, 1870 (2016).

# 28 Stamatakis, A. RAxML version 8: a tool for phylogenetic analysis and post-analysis of large phylogenies. Bioinformatics 30, 1312-1313 (2014).

# 29 Yang, Z. Yang ZH.. PAML 4: Phylogenetic analysis by maximum likelihood. Mol Biol Evol 24: 1586-1591. 24, 1586-1591 (2007).

# 30 Krogh, A., Larsson, B., Von, H. G. & Sonnhammer, E. L. Predicting transmembrane protein topology with a hidden Markov model: application to complete genomes. Journal of Molecular Biology 305, 567-580 (2001).

# 31 Lalitha, S. Primer Premier 5. Biotech Software & Internet Report 1, 270-272 (2000).
